# Effects of Lattice Parameters in Material-Extruded PLA Crank Arms on the Kinematics and Bearing Vibrations of an Inverted Slider-Crank Mechanism

**DOI:** 10.3390/polym18192335

**Published:** 2026-09-24

**Authors:** Caglar Sevim

**Affiliations:** Department of Mechanical Engineering, Faculty of Engineering, Niğde Ömer Halisdemir University, Niğde 51240, Türkiye; caglar.sevim@ohu.edu.tr

**Keywords:** poly(lactic acid), material extrusion, additive manufacturing, lattice structures, inverted slider-crank mechanism, vibration analysis, dynamic analysis

## Abstract

This study investigates the kinematic and vibration performance of an inverted slider-crank mechanism incorporating poly(lactic acid) (PLA) lattice crank arms fabricated by material extrusion (MEX). Gyroid, Schwarz-D, X, and 2.5X topologies with 5 and 10 mm cell sizes and nominal infill densities of 25%, 50%, and 75% were compared with aluminum and fully solid PLA reference crank arms. All 25% specimens failed before steady-state operation; comparisons therefore covered 16 configurations at 50% and 75%. Output-link angular acceleration, slider relative linear acceleration, and vibration levels at bearings $A_{0}$ and $B_{0}$ were measured. A rigid-body dynamic model solved using the fourth-order Runge–Kutta method provided a theoretical baseline. Increasing nominal infill from 50% to 75% reduced the average angular and linear acceleration root mean square error (RMSE) relative to aluminum by 66.2% and 76.0%, respectively; average bearing vibration reductions were 28.6% and 29.6%. Cell size showed no systematic trend. At 75% nominal infill, X and 2.5X crank arms combined low kinematic deviations and bearing vibration levels with approximately 55% lower mass than aluminum. These findings identify promising PLA lattice configurations for lightweight moving components under the investigated manufacturing and operating conditions.

## 1. Introduction

Additive manufacturing (AM) technologies have moved beyond the prototyping stage over the past decade and have become widely used in the production of final engineering components. Largely eliminating the geometric limitations of traditional machining methods, this technology provides high design freedom by allowing material to be placed only in regions required to carry mechanical loads [1,2,3,4]. This design freedom enables the realization of complex internal geometries that are difficult to produce using conventional methods, such as lattice structures; thus, the mass of parts can be reduced while optimizing their mechanical performance according to application requirements. Particularly in the aerospace industry, components developed with lattice structures and topology optimization are widely used due to the demand for a high strength-to-weight ratio [5,6,7,8].

Material extrusion (MEX), as classified in ISO/ASTM 52900:2021, is the additive manufacturing process category used in this study [9]. In filament-based MEX of thermoplastics such as poly(lactic acid) (PLA), the polymer is heated, extruded through a nozzle, and deposited along prescribed paths to form successive layers. Bond formation between deposited layers depends on polymer chain mobility and interdiffusion under the thermal history imposed by deposition and cooling [10]. Consequently, the mechanical performance of a printed polymer component reflects both its designed geometry and the structure developed during processing. For PLA, nozzle temperature, printing speed, layer thickness, and deposition direction have been shown to influence tensile properties and interlayer bonding [11]. These relationships are relevant to lattice crank arms, where the deposited polymer forms thin walls or struts that transmit repeated loads during mechanism operation. Their assessment therefore requires consideration of polymer processing alongside lattice geometry and component-level performance.

With the development of design and manufacturing capabilities provided by AM, the investigation of the mechanical and dynamic behavior of cellular materials, and particularly triply periodic minimal surface (TPMS)-based lattice structures, has become an important research area [12,13]. The performance and functional characteristics of strut-based (Octet, Grid, X-Lattice, etc.) and surface-based (Gyroid, Schwarz-D, Diamond) lattice structures are significantly affected by parameters such as unit cell topology, relative density, and characteristic geometric dimensions (strut diameter or wall thickness) [14,15,16,17]. Particularly, TPMS topologies such as Gyroid and Diamond can reduce sudden buckling and local collapse tendencies while providing high energy absorption by exhibiting progressive hardening behavior under load when appropriately designed [12,18]. In addition to mechanical properties, vibration transmission characteristics and dynamic responses can also be controlled by modifying the geometric properties of lattice structures, thus providing significant advantages in mitigating resonance-induced vibrations [19,20]. Studies on sandwich and hybrid lattice architectures also show that optimized topologies can reduce structural vibration levels and improve damping performance under dynamic loads [21,22].

From a machine dynamics perspective, the dynamic behavior of the system is affected by the mass and inertia properties of the links, as well as the structural flexibility and damping characteristics [23,24]. In the literature, numerous studies have been conducted to improve the dynamic behavior of mechanical systems by investigating link flexibility [25,26,27], friction, clearance, and wear effects in joints [28,29,30,31], the effects of lubrication conditions on dynamic behavior [32,33], and the effects of variable load conditions and load uncertainties on dynamic behavior [34,35].

Experimental studies investigating mechanism vibrations have demonstrated that factors such as joint clearances, link flexibility, and bearing conditions significantly influence dynamic response. Erkaya and Uzmay investigated the effects of joint clearance and link flexibility on the vibration behavior of a slider-crank mechanism, showing that link flexibility can mitigate additional vibrations caused by joint clearance [36]. Meanwhile, Ghazwani and Pham experimentally examined the vibration characteristics of a slider-crank mechanism under various bearing fault conditions, comparing the frequency-domain responses of healthy and damaged operating conditions [37]. Furthermore, the internal geometry of PLA structures fabricated by MEX has been shown to influence dynamic behavior. Öteyaka et al. reported that infill patterns and infill densities significantly alter the vibration damping properties of PLA specimens fabricated by MEX [38]. Similarly, Nisamudhin et al. investigated the vibration and damping behaviors of solid and lattice PLA beams fabricated by MEX using experimental, analytical, and finite element methods [39].

Studies on the application of additive manufacturing to moving mechanism links demonstrate the potential of this design approach in terms of mechanical and structural performance [40,41,42,43]. Furthermore, the mechanical properties, fatigue behavior, and energy absorption capacities of additively manufactured structures have been extensively investigated in the literature [44,45,46,47,48,49]. In our previous work, we examined the effects of manufacturing and design parameters—such as infill density, raster angle, and geometric thickness—on the dynamic responses and bearing vibrations of slider-crank and inverted slider-crank mechanisms utilizing crank arms fabricated by MEX [50,51]. However, those studies focused on conventional infill and cross-sectional parameters, without systematically evaluating the influence of different lattice topologies and cell sizes on the kinematic and vibrational behavior of a moving mechanism link. Similarly, the majority of research investigating the vibration and damping characteristics of lattice structures has been conducted on stationary specimens, beams, or static structures, with limited attention paid to their behavior as moving, load-bearing links within an actual mechanism.

Addressing this research gap, this study experimentally evaluates how lattice design affects the functional performance of PLA crank arms fabricated by MEX when used as moving, load-bearing components in an inverted slider-crank mechanism. Crank arms were produced with four lattice topologies—Gyroid (Gy), Schwarz-D (ScD), 2.5X-structure (2.5X), and X-structure (X)—at 5 and 10 mm cell sizes (CSs) and nominal infill densities (IDs) of 25%, 50%, and 75%. The lattice crank configurations were designated according to the topology–infill density–cell size sequence; for example, the code Gy-75-5 represents the Gyroid crank configuration with a 75% ID and a 5 mm CS. The obtained results were compared with an aluminum reference crank (AL-REF) and a 100% solid additively manufactured crank (AM-100). In the kinematic evaluation, in addition to the angular motion of the output link, the relative linear motion of the slider on the output link was also directly measured experimentally using a non-contact linear encoder. The obtained kinematic results were compared with the theoretical model; furthermore, bearing vibrations were measured to reveal the effects of different lattice structure parameters on the kinematic and dynamic behavior of the mechanism. Comparison with AM-100 evaluates the introduction of lattice architectures into a PLA crank arm, while comparison with AL-REF provides a benchmark for assessing lightweight polymer alternatives to the metallic component.

## 2. Inverted Slider-Crank Mechanism

The inverted slider-crank mechanism is one of the fundamental mechanism types that converts the rotational motion of the input crank into oscillatory motion of the output link. In this system, motion transmission is provided by a slider pivoted to the end of the crank and sliding along the output link. The kinematic diagram of the mechanism used in this study is presented in Figure 1.

Here, $A_{0}$ represents the center of rotation of the crank, and $B_{0}$ represents the pivot point of the output link. Depending on the angular position $\varphi_{21}$ of the crank arm (link number: 2) about $A_{0}$, the position of point A and consequently the magnitude of the position vector ($\vec{{AB}_{0}}$) continuously change. The loop-closure equation of the mechanism is expressed in vector form as follows:

(1) $$r_{A_{0}A}=r_{A_{0}B_{0}}+r_{B_{0}A}$$

When the scalar components of this vector relationship are expressed in terms of the crank angle $\varphi_{21}$ and the output link angle $\varphi_{41}$, the fundamental relationships used in the position analysis of the mechanism are obtained. Under the ideal rigid-body assumption, the coordinates of the centers of mass of the links and their angular position expressions are defined as follows:

(2) $$x_{G_{2}}=A_{0}G_{2}\cdot cos(\varphi_{21})$$

(3) $$y_{G_{2}}=A_{0}G_{2}\cdot sin(\varphi_{21})$$

(4) $$x_{G_{4}}=A_{0}B_{0}-G_{4}B_{0}\cdot cos(\varphi_{41})$$

(5) $$y_{G_{4}}=G_{4}B_{0}\cdot sin(\varphi_{41})$$

Due to the geometric constraints of the mechanism, the distance $AB_{0}$ and the output link angle ($\varphi_{41}$) are calculated using the following trigonometric relationships:

(6) $$AB_{0}=\sqrt{\left(A_{0}{A)}^{2}+{\left(A_{0}B_{0}\right)}^{2}-2(A_{0}A)\cdot (A_{0}B_{0})\cdot cos(\varphi_{21}\right)}$$

(7) $$\varphi_{41}=\sin^{-1}\left(\frac{A_{0}A\cdot \sin\left(\varphi_{21}\right)}{AB_{0}}\right)$$

Here, $G_{2}$ represents the center of mass of the crank arm, and $G_{4}$ represents the center of mass of the output link. $\varphi_{41}$ is the angular position of the output link with respect to the x-axis. The first and second derivatives of these position expressions, defined in terms of the parameter $\varphi_{21}$, were taken with respect to time to obtain the velocity and acceleration expressions, respectively.

The equations of motion for the dynamic analysis of the mechanism were derived using Lagrange’s method. By defining the total kinetic energy $\left(E_{kin}\right)$ and potential energy ($E_{pot}$) of the system, the Lagrangian function $\left(L=E_{kin}-E_{pot}\right)$ was formed. The equation of motion of the mechanism was obtained using the following Lagrange equation, considering the generalized coordinate $\varphi_{21}$ and the corresponding generalized force/moment (Q):

(8) $$\frac{d}{dt}\cdot \left[\frac{\partial L\left(\varphi_{21}\right)}{\partial {\dot{\varphi }}_{21}}\right]-\frac{\partial L\left(\varphi_{21}\right)}{\partial \varphi_{21}}=Q_{top}\left(\varphi_{21}\right)\;$$

The resulting second-order nonlinear differential equation system was numerically solved in MATLAB (R2019b, MathWorks, Natick, MA, USA) using the fourth-order Runge–Kutta (RK4) algorithm. The geometric dimensions of the mechanism used in the dynamic model and the mass and inertia parameters of the links are given in Table 1.

To evaluate the extent to which the experimental setup represents the theoretical behavior of the mechanism, the kinematic results obtained from the theoretical model based on the ideal rigid-body assumption were compared with the experimental measurements obtained using AL-REF. In this context, the angular position, velocity, and acceleration of the output link and the relative position, velocity, and acceleration of the slider were evaluated.

Figure 2a–c shows the variations in the angular position ($\varphi_{41}$), angular velocity (${\dot{\varphi }}_{41}$) and angular acceleration (${\ddot{\varphi }}_{41})$; Figure 2d–f shows the variations in the relative position ($x_{43}$), relative velocity (${\dot{x}}_{43}$) and relative acceleration (${\ddot{x}}_{43}$) of the slider on the output link.

As shown in Figure 2a,b, the experimental results regarding the angular position and velocity of the output link show a high degree of agreement with the theoretical model throughout one crank revolution. Similarly, in the relative position and velocity variations in the slider given in Figure 2d,e, the experimental and theoretical curves largely coincide.

However, more significant differences emerge in the acceleration results compared to the position and velocity results. In the angular acceleration of the output link [Figure 2c], although the experimental and theoretical results show a similar overall cyclic variation pattern, significant deviations are observed, especially at the beginning and end of the cycle, in terms of amplitude and peak positions. In contrast, in the relative acceleration results of the slider [Figure 2f], the agreement between the theoretical and experimental curves is higher, and only local fluctuations and limited amplitude differences are observed at some crank angles. These deviations in the acceleration data are considered to be associated with effects that are not taken into account in the ideal rigid-body model, such as joint clearances, friction, limited flexibility of the connecting elements, and amplification of measurement noise during the numerical differentiation of the experimental position data.

Overall, the high agreement obtained in the position and velocity results, along with the similarity in the acceleration behavior of the slider, indicates that the experimental setup adequately represents the fundamental kinematic behavior of the ideal mechanism. The deviations observed in the angular acceleration of the output link, however, reveal the dynamic differences between the ideal rigid-body model and the actual experimental system. This validation shows that the experimental system can be used as a reliable reference for comparing the effects of different lattice crank configurations on the kinematic and dynamic behavior of the mechanism.

## 3. Materials and Methods

The input parameters examined in the study and the output parameters used to evaluate the mechanism’s performance are summarized in Table 2.

### 3.1. Experimental Setup and Instrumentation

In this study, an experimental test setup was created to systematically investigate the effects of lattice-structured crank arms on the kinematic and dynamic behavior of an inverted slider-crank mechanism. The general structure of the experimental setup and sensor placements are shown in Figure 3. The system consists of the mechanism body, drive system [Figure 3a], vibration measurement sensors [Figure 3b,c], and kinematic measurement sensors [Figure 3b,d,e]. The mechanism was driven by a 1.5 kW asynchronous motor coupled to a gearbox with a 10:1 reduction ratio, and the motor speed was controlled by a motor drive. All experiments were performed at a constant crankshaft speed of 118.4 rpm.

The National Instruments (NI) USB-6251 data acquisition system (National Instruments Corporation, Austin, TX, USA) was used to collect the kinematic measurement data. The angular positions of the crank and output links were measured by means of rotary encoders with resolutions of 1024 pulses/rev and 2000 pulses/rev, respectively, mounted on the crank and output shafts [Figure 3b,d]. The relative linear position of the slider on the output link was determined by means of a magnetic linear scale placed along the output link and a non-contact linear magnetic encoder with a resolution of 10 µm [Figure 3e]. Owing to the non-contact measurement principle, the relative position data were obtained without introducing any additional contact force or friction into the motion of the slider.

To measure the vibrations occurring in the bearing regions of the mechanism, Brüel & Kjær Type 4514-B uniaxial accelerometers (Brüel & Kjær Sound & Vibration Measurement A/S, Nærum, Denmark) were used, with separate accelerometers placed in the X and Y directions at bearings $A_{0}$ and $B_{0}$ [Figure 3b,c]. The vibration signals obtained from the accelerometers were recorded through separate measurement channels using the Brüel & Kjær Type 3560 B data acquisition system (Brüel & Kjær Sound & Vibration Measurement A/S, Nærum, Denmark).In the experimental system, a specially developed trigger unit was used to initiate the recording of the NI USB-6251 and Brüel & Kjær data acquisition systems and the start command of the motor driver using a common trigger signal. The trigger signal simultaneously initiated data recording for the kinematic and vibration measurement systems and sent the start command to the motor driver. The general flowchart of the experimental measurement and data acquisition system is presented in Figure 4.

### 3.2. Design and Material Extrusion of PLA Lattice Crank Arms

The geometric dimensions and details of the designed crank arm are given in Figure 5. To ensure sufficient rigidity and strength in the joint regions at points A and $A_{0}$ of the crank arm, these regions were designed as fully solid. Four different lattice topologies—Gyroid, Schwarz-D, 2.5X-structure, and X-structure—were applied to the 100 mm long central region shown as the shaded area in Figure 5. Each topology was modeled using 5 mm and 10 mm CSs and nominal ID levels of 25%, 50%, and 75%, corresponding to “Solidity” values of 0.25, 0.50, and 0.75, respectively, within the Volumetric Lattice tool of Autodesk Fusion software (version 2704.1.36). These values do not represent the infill setting in the slicing software or the experimentally determined actual relative densities of the printed specimens.

The unit cell views of the lattice topologies used in the study are given in Figure 6. Gy and ScD are surface-based TPMS topologies, while X and 2.5X are strut-based lattice topologies. Crank geometries were created using Autodesk Fusion (version 2704.1.36, Autodesk Inc., San Francisco, CA, USA). The resulting models were exported in stereolithography (STL) format and transferred to Ultimaker Cura (version 5.10.1, UltiMaker, Utrecht, The Netherlands), where the corresponding G-code files were generated. The crank arms were fabricated from commercial CR Series poly(lactic acid) (PLA) filament with a nominal diameter of 1.75 mm (Creality, Shenzhen Creality 3D Technology Co., Ltd., Shenzhen, China). The manufacturer reports a density of 1.24 g/cm^3^, a tensile strength of 51 MPa, and a flexural strength of 86 MPa for this material [52].

The specimens were manufactured by filament-based MEX using a Bambu Lab X1 Carbon Combo 3D printer equipped with a 0.4 mm nozzle (Bambu Lab, Shenzhen, China). The crank arms were printed flat on the build plate, with their longitudinal axes parallel to the plate and the joint-hole axes parallel to the build direction (Z-axis). The PLA filament was heated and extruded through the nozzle, deposited according to the sliced geometry, and allowed to solidify during cooling. The nozzle and build-plate temperatures were set to 220 °C and 60 °C, respectively. A layer height of 0.2 mm, a line width of 0.4 mm, and a printing speed of 200 mm/s were specified. The manufacturing parameters and manufacturer-reported PLA properties are summarized in Table 3. A sufficiently high wall count was specified in the slicing software so that the material regions of the lattice computer-aided design (CAD)models were printed entirely using wall paths, without separate infill paths. This setting describes the deposition strategy within the modeled material regions; the nominal ID was determined by the CAD lattice geometry, as defined above, rather than by the slicer infill percentage.

The general appearances of the crank specimens produced using different lattice topologies, CS, and IDs are shown in Figure 7.

A total of eight specimens were tested—one from each of the eight configurations comprising four lattice topologies and two CS values—at 25% ID. In the experiments, it was observed that all these specimens fractured during the first revolution of the crankshaft or at the beginning of the second revolution. The fractures occurred approximately in the middle sections of the central lattice region. Representative photographs of the fractures in the Gyroid specimens are presented in Figure 8. This observation indicated that the crank arms with 25% ID failed to provide sufficient mechanical strength to withstand the dynamic loads under the given experimental conditions. Since the specimens fractured before reaching a steady operating state, kinematic and dynamic data suitable for comparative analysis could not be obtained; consequently, further analyses were conducted on specimens with 50% and 75% ID.

At low ID levels, the transfer of the load through more limited load-bearing cross-sections may have increased local stresses within the lattice elements, thereby contributing to premature failure. Variations in cross-section and stiffness between the solid connection regions and the lattice region could also lead to local stress concentrations. However, as the fracture locations observed in the experiments were situated within the central lattice region, stress concentration at the transition region was not identified as the confirmed primary cause of these failures. Since the local stress distribution and crack initiation were not specifically determined, this explanation is offered as a possible mechanism.

The masses of the produced crank arms were measured, and the obtained values are given in Table 4. While the masses of specimens with 5 mm and 10 mm CSs at the same lattice topology and ID were quite similar, it was observed that the mass values varied depending on the lattice topology and ID.

The material volumes ($V_{L}$) of the central lattice regions of the cranks with nominal ID values of 50% and 75% used in the comparative analyses were obtained from the volume data in Autodesk Fusion software. The reference volume of the central region in its fully solid state was determined as $V_{0}$ = 45,000 mm^3^ using the dimensions of 100 mm × 15 mm × 30 mm. The CAD-based geometric volume ratio was calculated using the following equation:

Geometric volume ratio (%) = (*V_L_*/*V*_0_) × 100

The resulting CAD volumes and geometric volume ratios are presented in Table 4, together with the crank masses measured using a precision balance. The volume ratios pertain to the CAD geometry, whereas the mass measurements apply to the entire crank, including the solid connection regions. The calculated volume ratios were not considered as actual post-production relative density measurements of the printed specimens.

### 3.3. Experimental Procedure and Data Processing

To ensure the same starting position in all experiments, the crank arm was brought to the 0° starting position using a digital protractor before each experiment [Figure 3e]. Kinematic data were recorded in LabVIEW at a sampling rate of 10 kS/s, with 1000 samples per data acquisition cycle. Kinematic recordings covering multiple operating cycles of the mechanism were obtained for each crank configuration. To avoid the influence of the initial transient operating region on the results, the analyses were performed over one complete crank cycle selected from the portion of the recording in which the mechanism had reached a stable operating state. The same cycle selection procedure was applied in all experiments, and the angular position of the output link and the relative linear position of the slider corresponding to one complete rotation of the crankshaft from 0° to 360° were evaluated. For the comparative analyses, one physical specimen was used for each crank configuration, and no experimental repetitions were performed for the kinematic measurements.

Angular and linear position data obtained from the encoders were processed in MATLAB. Angular and linear velocity values were calculated by taking the numerical derivative of the unfiltered position data with respect to time using the “gradient” function. To reduce the high-frequency fluctuations resulting from numerical differentiation, a triangular weighted moving average filter with a window width of 100 data points was applied to the velocity data. Acceleration values were then obtained by taking the numerical derivative of the filtered velocity data, and the same filtering process was applied to the acceleration data. The window, which is approximately 10 ms and corresponds to about 2% of one crank revolution, was selected to emphasize the fundamental cyclic kinematic response, although it may also attenuate high-frequency components associated with joint clearances, impacts, and local vibrations.

Root mean square error (RMSE) values were calculated to quantitatively compare the kinematic responses of lattice-structured crank arms with the reference cranks. The angular acceleration of the output link (${\ddot{\varphi }}_{41}$) and the relative linear acceleration of the slider (${\ddot{x}}_{43}$) for each lattice configuration were separately compared with the experimental acceleration responses obtained from the AL-REF crank and the AM-100 crank. The compared signals were aligned to a common angular axis over the crank angle range of 0–360°, and the RMSE value was calculated using the following equation:

(9) $$RMSE=\sqrt{\frac{1}{N}\sum_{i=1}^{N}{\left[y_{L,i}-y_{ref,i}\right]}^{2}}$$

Here, $y_{L,i}$ represents the acceleration value obtained from the lattice-structured crank configuration, $y_{ref,i}$ represents the acceleration value obtained from the AL-REF or AM-100 crank, and *N* represents the number of data points used in the comparison. RMSE values were calculated separately for the angular acceleration of the output link and the relative linear acceleration of the slider, and a lower RMSE value indicates that the lattice configuration is closer to the reference kinematic response.

Vibration recordings were performed in the Brüel & Kjær PULSE environment, and the vibration data obtained from the Overall Analyzer were used in the evaluation. The frequency range of the Overall Analyzer was set to 800 Hz, with an exponential averaging time of 1/8 s and a time constant of 1/16 s. The sampling rate of the exported vibration level series used for the PSD calculation was determined to be approximately 100 Hz based on the median of the time steps. No additional filtering or smoothing was applied in MATLAB to the vibration data exported from the PULSE environment.

Vibration measurements were performed in two separate experimental runs on the same physical specimen for each crank configuration. The vibration evaluation procedures described below were applied separately to both experimental runs. A stable operating interval of 6–8 s, away from the initial and transient regions, was used as the common analysis window for all configurations.

To evaluate the vibration levels at bearings $A_{0}$ and $B_{0}$ by considering both measurement directions, the resultant vibration levels were calculated from the vibration values obtained in the X and Y directions. The resultant vibration levels for each data point were determined using the following equations:

(10) $$a_{A_{0},i}=\;\sqrt{a_{A_{0}X,i}^{2}+a_{A_{0}Y,i}^{2}}$$

(11) $$a_{B_{0},i}=\sqrt{a_{B_{0}X,i}^{2}+a_{B_{0}Y,i}^{2}}$$

The bearing vibration level for each experimental run was calculated by averaging the resultant values obtained over the stable operating interval of 6–8 s:

(12) $${\bar{a}}_{A_{0}}=\frac{1}{N}\sum_{i=1}^{N}a_{A_{0},i}$$

(13) $${\bar{a}}_{B_{0}}=\frac{1}{N}\sum_{i=1}^{N}a_{B_{0},i}$$

Here, $a_{A_{0}X,i}$ and $a_{A_{0}Y,i}$ represent the vibration values obtained in the X and Y directions of bearing $A_{0}$; $a_{B_{0}X,i}$ and $a_{B_{0}Y,i}$ represent the vibration values obtained in the X and Y directions of bearing $B_{0}$. *N* is the number of data points in the 6–8 s analysis interval.

The final bearing vibration levels representing each crank configuration were determined by taking the arithmetic mean of the average values obtained from the two experimental runs:

(14) $${\bar{a}}_{A_{0},rep}=\frac{{\bar{a}}_{A_{0_{1}}}+{\bar{a}}_{A_{0_{2}}}}{2}$$

(15) $${\bar{a}}_{B_{0},rep}=\frac{{\bar{a}}_{B_{0_{1}}}+{\bar{a}}_{B_{0_{2}}}}{2}$$

Here, subscripts (1) and (2) represent the vibration levels obtained from the two separate experimental runs.

In addition to the vibration evaluation in the time domain, power spectral density (PSD) analysis was performed to examine the frequency content of the selected crank configurations. In the PSD calculations, the same 6–8 s steady-state operating interval was used to ensure consistency with the vibration analyses in the time domain. For each experimental run, the mean values of the resultant vibration time series for bearings $A_{0}$ and $B_{0}$ were removed, and PSD estimation was performed using the Welch method. The analysis employed a periodic Hann window with a length of 100 points (corresponding to 1 s), 50% overlap, and a 512-point fast Fourier transform (FFT). Three overlapping segments were obtained for each experimental run from the selected 2 s interval. The FFT frequency step was 0.1953 Hz, and the equivalent noise bandwidth of the window was 1.5 Hz. The PSD was calculated from the time series of the resultant overall vibration level. Comparisons around the 1X component were performed at 1.953125 Hz, which is the frequency bin closest to 1.973 Hz, and the results in the frequency domain were evaluated considering the 1X, 2X, and 3X harmonics. For presentation purposes, the PSD curves were displayed in displayed in decibels (dB) using the 10log_10_ (PSD) transformation, while percentage changes were calculated from the linear PSD values. For the purpose of comparing the first three rotational components, the results are presented in the frequency range of 0–10 Hz.

## 4. Results

### 4.1. Kinematic Acceleration Responses

To evaluate the effects of lattice structure parameters on the kinematic behavior of the mechanism, the angular acceleration of the output link (${\ddot{\varphi }}_{41}$) and the relative linear acceleration of the slider (${\ddot{x}}_{43}$) were investigated. Experimental acceleration curves for each lattice topology at 50% and 75% IDs and 5 mm and 10 mm unit CSs were compared with the experimental results obtained from the AL-REF and AM-100 cranks and the theoretical results obtained using the RK4 method. The results for the Gy, ScD, 2.5X, and X topologies are presented in Figure 9, Figure 10, Figure 11 and Figure 12, respectively.

When Figure 9, Figure 10, Figure 11 and Figure 12 are considered together, it can be seen that the ID has a clear effect on the kinematic acceleration behavior of the mechanism. Crank arms with a 50% ID in all lattice topologies exhibited pronounced acceleration oscillations compared to the reference curves, especially in the crank angle range of 0–150°, both in the angular acceleration of the output link [Figure 9, Figure 10, Figure 11 and Figure 12a,b] and in the relative linear acceleration of the slider [Figure 9, Figure 10, Figure 11 and Figure 12c,d]. It is observed that these oscillations decreased when the ID was increased to 75%, and the obtained kinematic responses became closer to the experimental curves obtained from the AL-REF and AM-100 cranks. This result shows that a higher ID reduces the effect of lattice-structured crank arms on the kinematic response of the mechanism.

When comparing the results at a 50% ID across topologies, the Gy, ScD, and X topologies showed higher acceleration oscillations, particularly in the initial part of the cycle, while the 2.5X topology exhibited lower-amplitude oscillations, providing responses closer to the reference kinematic behavior. At a 75% ID, deviations decreased in all topologies, and the differences between topologies narrowed considerably.

Figure 13 shows the RMSE values of the kinematic acceleration responses of lattice-structured crank configurations calculated with respect to the AL-REF and AM-100 references. The results show that increasing the ID from 50% to 75% reduces the deviation in the kinematic acceleration responses for all topology and CS combinations. In all eight matched configurations with the same topology and CS, a 75% ID provided lower RMSE values for both the angular acceleration of the output link and the relative linear acceleration of the slider.

The mean RMSE values according to ID are summarized in Table 5 and represent the arithmetic mean of the RMSE values of eight different configurations consisting of four topologies and two CS values at each ID level. According to AL-REF, the angular acceleration RMSE value of the output link decreased from 27.93 $rad/s^{2}$ at 50% ID to 9.44 $rad/s^{2}$ at 75% ID, resulting in a decrease of approximately 66.2%. Similarly, the RMSE of the slider relative linear acceleration decreased from 20.08 $m/s^{2}$ to 4.83 $m/s^{2}$, representing a decrease of approximately 76.0%. Based on the AM-100 reference, a decrease of 76.5% was observed for the output link and 79.9% for the slider in the average RMSE values.

When the topologies were evaluated separately, the 2.5X-50-5 and 2.5X-50-10 configurations yielded lower RMSE values for both kinematic responses compared to the other 50% ID configurations. At 75% ID, the X-75-5, X-75-10, 2.5X-75-5, and 2.5X-75-10 configurations formed the group that yielded the lowest RMSE values with respect to both references. Increasing the CS from 5 mm to 10 mm did not lead to a uniform change in kinematic deviations across all topologies and showed a less systematic effect compared to the ID.

### 4.2. Vibration Responses

The effect of lattice structure parameters on bearing vibrations was investigated through vibration levels measured in the X and Y directions in bearings $A_{0}$ and $B_{0}$. Resultant vibration levels were calculated from the measurements obtained in both bearings, and the average values in the 6–8 s steady-state operating range were compared with the reference values for the AL-REF and AM-100 cranks. In Figure 14, the columns show the average vibration levels obtained from two experimental runs (*n* = 2), and the error bars indicate the minimum–maximum range of the two run averages.

It was observed that the ID had a clear effect on the vibration level in both bearings. In all configurations with the same topology and CS, increasing the ID from 50% to 75% reduced the resultant vibration level in both $A_{0}$ and $B_{0}$ bearings. Based on the average values of two experimental runs, the average vibration reduction for the eight paired configurations examined was determined to be approximately 28.6% in the $A_{0}$ bearing and 29.6% in the $B_{0}$ bearing. Furthermore, this reduction trend was maintained in all eight paired configurations in both experimental runs. When evaluated in terms of CS, configurations with a 5 mm CS showed lower vibration levels than those with a 10 mm CS in most cases, although this trend was not consistent across all topologies and IDs. The lowest average vibration level in the $A_{0}$ bearing was obtained in the X-75-5 configuration as 0.814 $m/s^{2}$; this value was found to be 22.1% lower than AL-REF and 2.4% lower than AM-100. In the $B_{0}$ bearing, the lowest average vibration level was measured at 1.154 $m/s^{2}$ in the 2.5X-75-5 configuration; this represents a vibration level 22.9% and 12.3% lower than the AL-REF and AM-100 references, respectively. The fact that the lowest vibration levels were obtained in different configurations in the two bearings indicates that the effect of the lattice topology on the vibration response in the bearing regions can vary depending on the bearing location.

Figure 15 shows the time-dependent vibration responses measured in the $A_{0\;}$ and $B_{0}$ bearings of the X-50-5 and X-75-5 configurations, compared with the AL-REF and AM-100 reference cranks. Higher vibration levels and peak values were observed in the X-50-5 configuration, particularly in both directions of the $B_{0}$ bearing. Increasing the ID to 75% markedly reduced these levels in the X-75-5 configuration, resulting in levels generally close to or lower than those of the reference cranks. This behavior is consistent with the resultant vibration levels obtained in Figure 14. To evaluate the frequency-domain counterpart of the time-domain vibration results, PSD analysis was performed for the AL-REF and AM-100 references and the X-50-5, X-75-5, 2.5X-50-5, and 2.5X-75-5 configurations. The results are presented in Figure 16.

Figure 16 shows that the spectral content of the overall resultant vibration level is concentrated particularly around the fundamental rotational frequency of the mechanism, 1X (≈1.97 Hz). The linear PSD unit is (m/s^2^)^2^/Hz, and the reference value for the dB representation is 1 (m/s^2^)^2^/Hz. Increasing the ID from 50% to 75% in these configuration pairs markedly reduces the PSD values around 1X in both bearings. In the X topology, the linear PSD value around 1X decreased by approximately 96.2% in bearing $A_{0}$ and 99.5% in bearing $B_{0}$ with the transition from 50% to 75% ID. Similarly, in the 2.5X topology, the same decrease was determined to be approximately 79.0% and 83.0%, respectively. The changes around the 2X and 3X harmonics showed a more limited and configuration-dependent behavior compared to the 1X component. These results indicate that the vibration reduction determined in the time domain occurs particularly in conjunction with the decrease in spectral content around the fundamental rotational frequency.

### 4.3. Combined Kinematic and Vibration Assessment

Kinematic performance was evaluated using RMSE values calculated for the angular acceleration of the output link and the relative linear acceleration of the slider, with AL-REF as the reference; vibration performance was evaluated using vibration levels measured in bearings $A_{0}$ and $B_{0}$. Figure 17 presents the combined effect of ID, lattice topology, and CS on the kinematic and vibration responses.

Figure 17 shows that increasing the ID from 50% to 75% reduces both the kinematic RMSE values and the bearing vibration levels in all examined lattice topology and CS combinations. This trend was observed in all eight paired design combinations, although the magnitude of the reduction varied depending on the topology. At 50% ID, the 2.5X topology yielded lower values in both kinematic and vibration responses compared to the other topologies, while at 75% ID, the differences between topologies decreased, with the X and 2.5X topologies particularly standing out in terms of both performance metrics.

As shown in Table 4, the same nominal ID level does not yield the same geometric volume ratio across different topologies. For instance, with a cell size of 5 mm and a nominal ID level of 75%, the geometric volume ratios were calculated as 75.29%, 80.20%, 92.70%, and 94.85% for the Gy, ScD, X, and 2.5X topologies, respectively. Therefore, differences in material quantity must also be taken into account when comparing the kinematic and vibration responses of the topologies. The results illustrate the behavior of crank configurations at the same nominal design levels; the effect of topology was not evaluated independently of variations in geometric volume ratio and mass.

The effect of CS did not show as systematic a change as the ID. At the same topology and ID, the direction and magnitude of the differences between 5 mm and 10 mm CSs varied depending on the configuration. This indicates that among the examined design parameters, the ID has a more consistent effect on the kinematic and dynamic responses, while the effect of CS should be evaluated in conjunction with the lattice topology and ID. It has been shown that geometric deviations in lattice elements produced by MEX can vary depending on element thickness and inclination angle [53]. When the cell size decreases, producing thinner elements with a constant line width can increase the relative effect of geometric deviations in local cross-sections. These manufacturing effects may contribute to variations in the influence of CS on the dynamic response, depending on topology and ID. Including mass reduction in this evaluation, a general performance comparison of the configurations at 75% ID is presented in Table 6.

The results show that mass reduction does not directly lead to better dynamic performance. Although Gy configurations achieved the highest mass reduction in the 75% ID group studied, they exhibited higher kinematic RMSE and bearing vibration levels. In contrast, at 75% ID, the X and 2.5X configurations with slightly higher mass achieved approximately 55% mass reduction compared to AL-REF and 3.0–3.2% compared to AM-100 while maintaining low kinematic deviations and bearing vibration levels.

This result demonstrates that maximum weight reduction alone is not a suitable design goal in lattice-structured crank design. When reducing the weight of a moving mechanism element, the resulting mass reduction must be evaluated together with its effects on the kinematic performance and bearing vibrations of the mechanism.

The reduction in kinematic deviations associated with an increase in nominal ID may be linked to the rise in effective stiffness—resulting from the increased amount of material in the load-bearing region—and the consequent limitation of elastic deformations under dynamic loads. However, the effects of changes in mass, mass moment of inertia, and stiffness on dynamic response differ from one another. While total mass affects inertial forces, the mass moment of inertia depends on the spatial distribution of the material relative to the axis of rotation. Therefore, the effects of mass and stiffness changes resulting from increased ID do not necessarily occur at the same rate or in the same direction in the dynamic response.

The effective stiffness of lattice structures depends on whether the load-bearing elements resist the load through axial tension–compression or bending. In structures dominated by tension–compression, transferring the load along the element axes can yield higher stiffness at the same relative density compared to structures where bending and element rotations dominate [54]. However, in TPMS structures, the contributions of in-plane tension–compression and bending vary depending on the geometry generation method, relative density, and loading conditions [55]. The fact that the linear load-bearing elements in the X and 2.5X geometries shown in Figure 6 transfer load components aligned with their axes directly via tension–compression can limit local bending and rotations at the junctions. This mode of load transfer, combined with the higher geometric volume ratios indicated in Table 4, may contribute to better preservation of the geometry between joints during operation and a reduction in kinematic deviations. Nevertheless, transverse load components acting on the crank arm must also be considered; the observed difference in performance cannot be attributed solely to axial stiffness. Damping, on the other hand, is a distinct mechanism associated with the dissipation of mechanical energy. Since a reduction in bearing vibrations may also stem from changes in excitation forces or force transmission, it is not, in isolation, an indicator of higher structural damping.

The lattice crank arms should also be considered as processed polymer structures. In printed PLA, interlayer bonding and process-induced voids can influence the mechanical response of the deposited material [11]. These small-scale voids are distinct from the intentionally designed openings of the lattice geometry. With the wall-path-only strategy used here, the contours of the sliced lattice sections govern the local arrangement and directions of the deposited roads. Changes in topology and cell size can therefore modify the deposition pattern within the load-bearing regions even when the nominal printing settings remain unchanged. This interaction between lattice design and polymer processing may contribute to the configuration-dependent behavior alongside the differences in geometric volume ratio and mass. However, the present measurements do not establish differences in interlayer bond quality or process-induced porosity between configurations; these remain possible contributors rather than experimentally confirmed explanations.

The ideal rigid-body model employed in this study is intended for comparison of the fundamental kinematic behavior with AL-REF and does not account for topology-specific variations in flexibility or damping of the lattice crank arms. No separate experimental measurements were conducted to determine the stiffness, damping ratios, natural frequencies, or elastic deformations of the lattice crank arms during operation. The physical explanations presented herein are regarded as potential mechanisms for interpreting the observed kinematic and vibration behaviors.

## 5. Conclusions

In this study, the effects of PLA crank arms fabricated by MEX with different lattice topologies, cell sizes, and nominal infill densities on the kinematic and vibration behavior of an inverted slider-crank mechanism were experimentally investigated. The main results are summarized below.

- All lattice cranks with a 25% ID fractured within the first 1–2 revolutions of the crankshaft and failed to reach a stable operating state. This result demonstrated that a 25% ID is insufficient to withstand dynamic loads under the current operating conditions.
- Increasing the ID from 50% to 75% reduced kinematic deviation in all topology and CS combinations examined. In all eight paired design configurations, a 75% ID yielded lower RMSE values for both the output link angular acceleration and the slider relative linear acceleration. According to AL-REF, the average RMSE reductions were determined to be 66.2% and 76.0%, respectively.
- In all eight paired configurations with the same topology and CS, a 75% ID reduced vibration levels in both $A_{0}$ and $B_{0}$ bearings; based on the averages of two experimental runs, the average reductions were calculated as 28.6% and 29.6%, respectively. The lowest average vibration level in the $A_{0}$ bearing was obtained as 0.814 $m/s^{2}$ in the X-75-5 configuration. In the $B_{0}$ bearing, the minimum average vibration level was determined as 1.154 $m/s^{2}$ in the 2.5X-75-5 configuration, with the X-75-5 configuration yielding a similar result of 1.240 $m/s^{2}$.
- In a comparison based on linear PSD values, the PSD value around 1X decreased by approximately 96.2% in the $A_{0}$ bearing and 99.5% in the $B_{0}$ bearing with the transition from X-50-5 to X-75-5. Similarly, in the comparison of 2.5X-50-5 and 2.5X-75-5, this decrease was determined to be approximately 79.0% and 83.0%, respectively.
- The effect of CS on kinematic and vibration behavior was not as systematic as that of the ID and varied depending on topology and ID. In particular, it was observed that the differences between 5 mm and 10 mm CSs decreased in many configurations, especially at 75% ID.
- At a 50% ID, the 2.5X topology showed lower kinematic RMSE values than the other lattice structures. At a 75% ID, the X and 2.5X topologies stood out as the design groups that achieved the lowest kinematic deviations. The X-75-5 and 2.5X-75-5 configurations, with a mass of approximately 114.5 g, achieved approximately 55% mass reduction compared to AL-REF while maintaining low kinematic RMSE and low bearing vibration levels.

These findings show that lattice design can be used to tailor the functional performance of PLA crank arms fabricated by MEX. For lightweight polymer moving components, reductions in material use and mass must be evaluated together with kinematic fidelity and bearing vibration behavior under the intended manufacturing and operating conditions.

These results are valid for the 118.4 rpm operating speed and experimental conditions examined in this study; the investigation of the effects of lattice parameters on dynamic behavior at different operating speeds may be considered a subject for future studies.

## Figures and Tables

**Figure 1:**
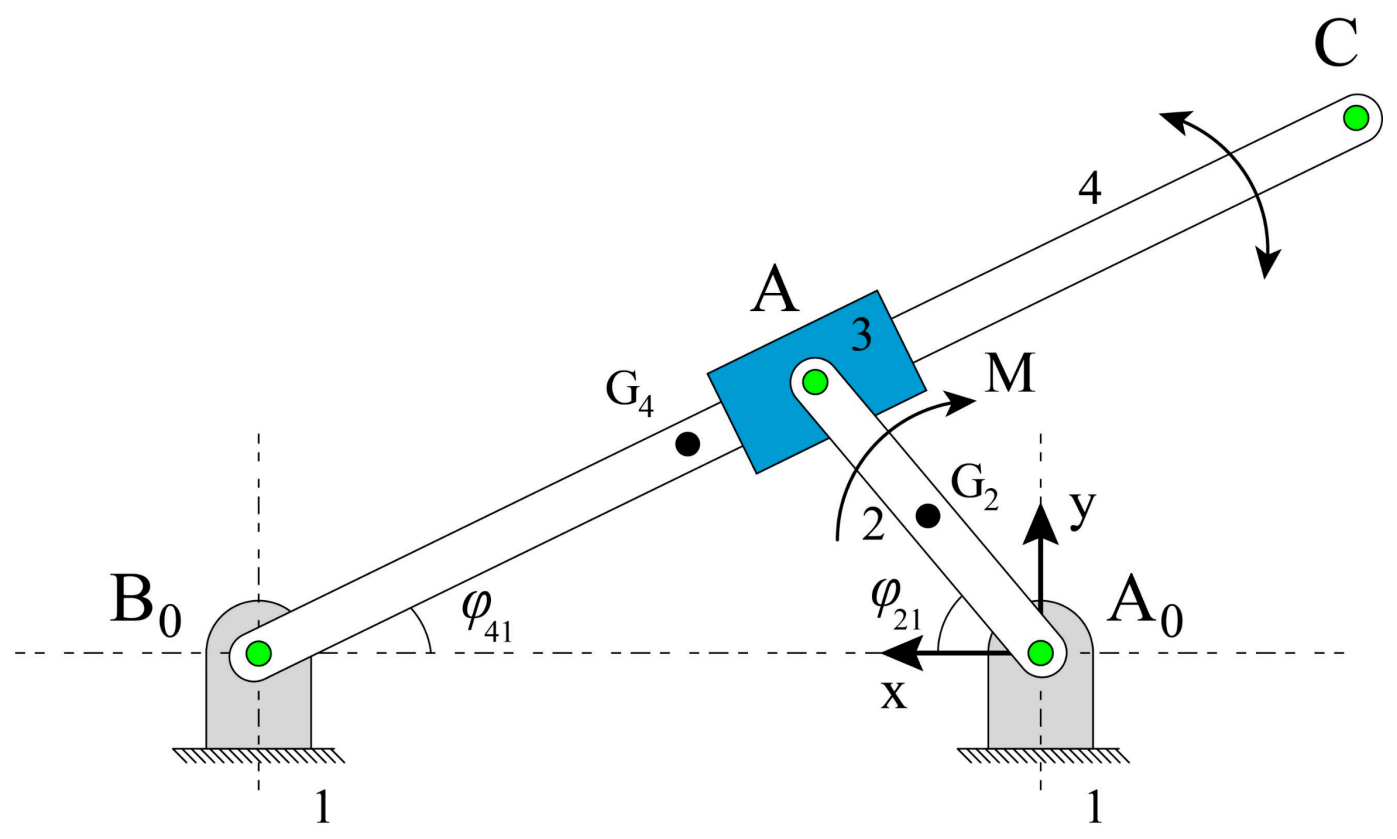
Schematic representation of an inverted slider-crank mechanism. Links 1–4 denote the fixed frame, crank, slider, and output link, respectively; arrows indicate the directions of rotation and the applied moment (*M*). Green circles indicate revolute joints.

**Figure 2:**
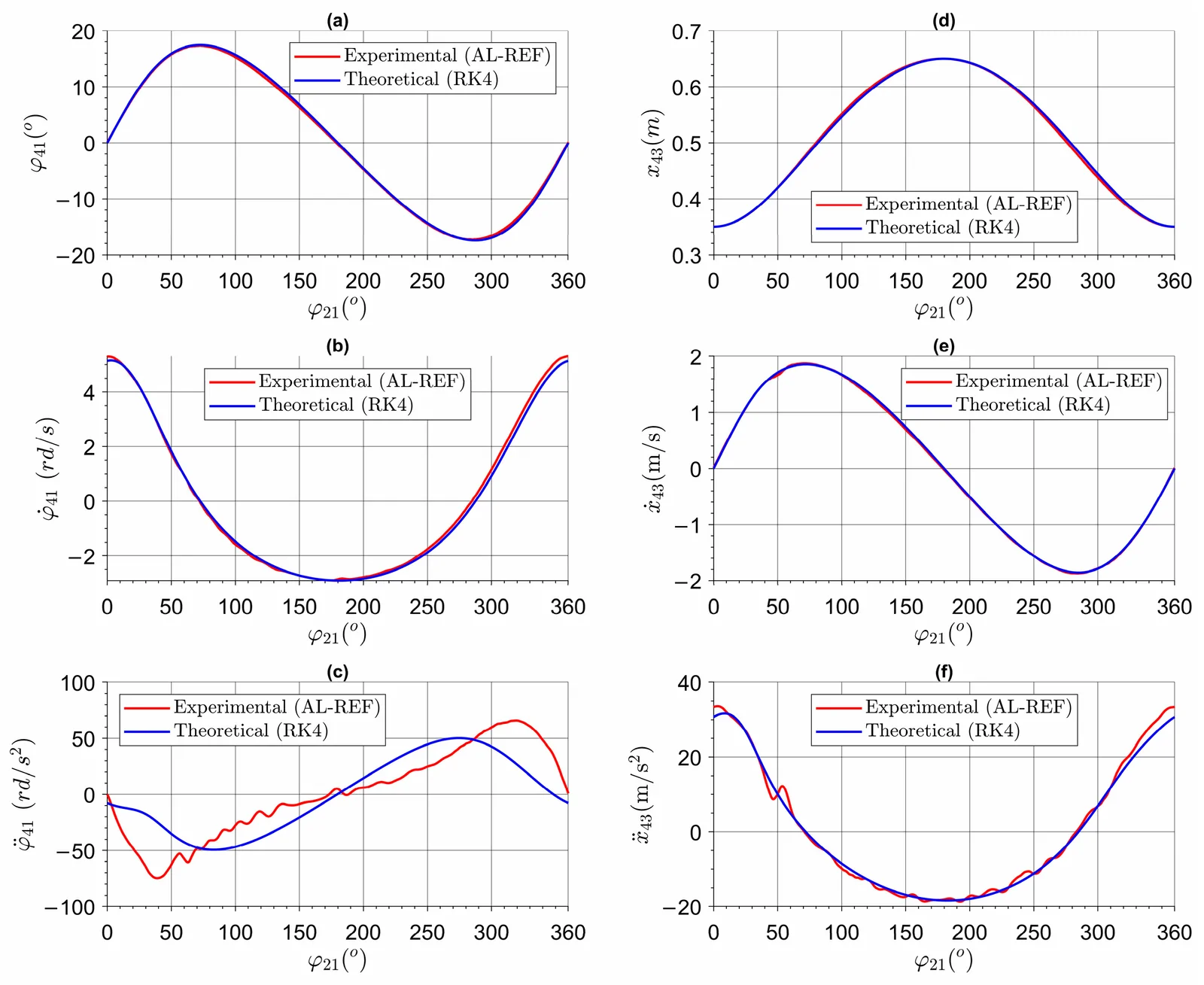
Comparison of theoretical and experimental kinematic results for the aluminum reference crank (AL-REF) with respect to crank angle ($\varphi_{21}$): output link angular position, angular velocity, and angular acceleration (**a**–**c**); relative position, velocity, and acceleration of the slider (**d**–**f**). RK4 denotes the fourth-order Runge–Kutta method.

**Figure 3:**
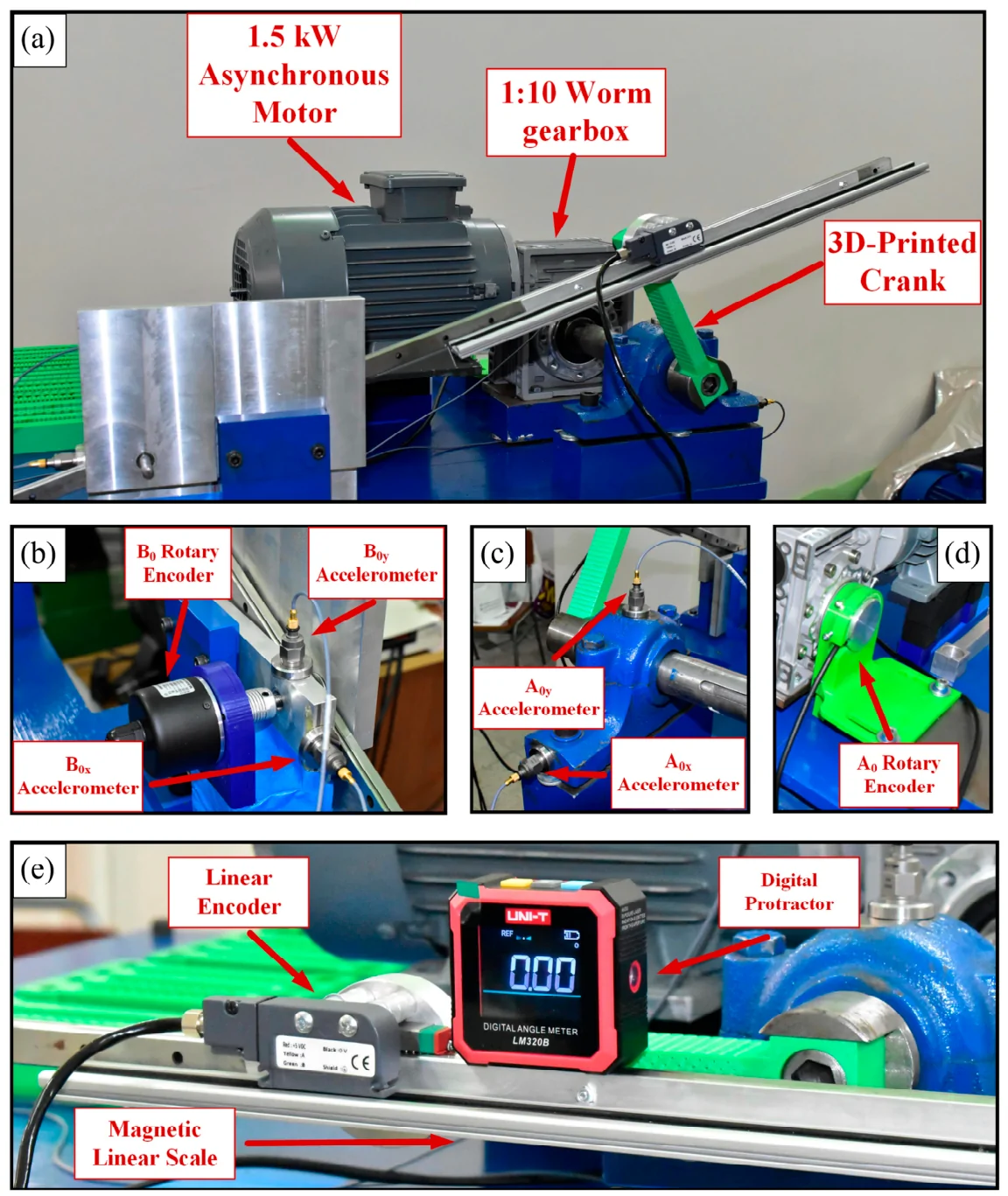
Experimental setup and instrumentation of the inverted slider-crank mechanism: (**a**) general view of the experimental setup, (**b**) $B_{0}$ output-shaft section with rotary encoder and accelerometers, (**c**) $A_{0}$ main bearing support with accelerometers, (**d**) $A_{0}$ crankshaft rotary encoder assembly, and (**e**) linear displacement measurement system and digital protractor used for initial angular positioning.

**Figure 4:**
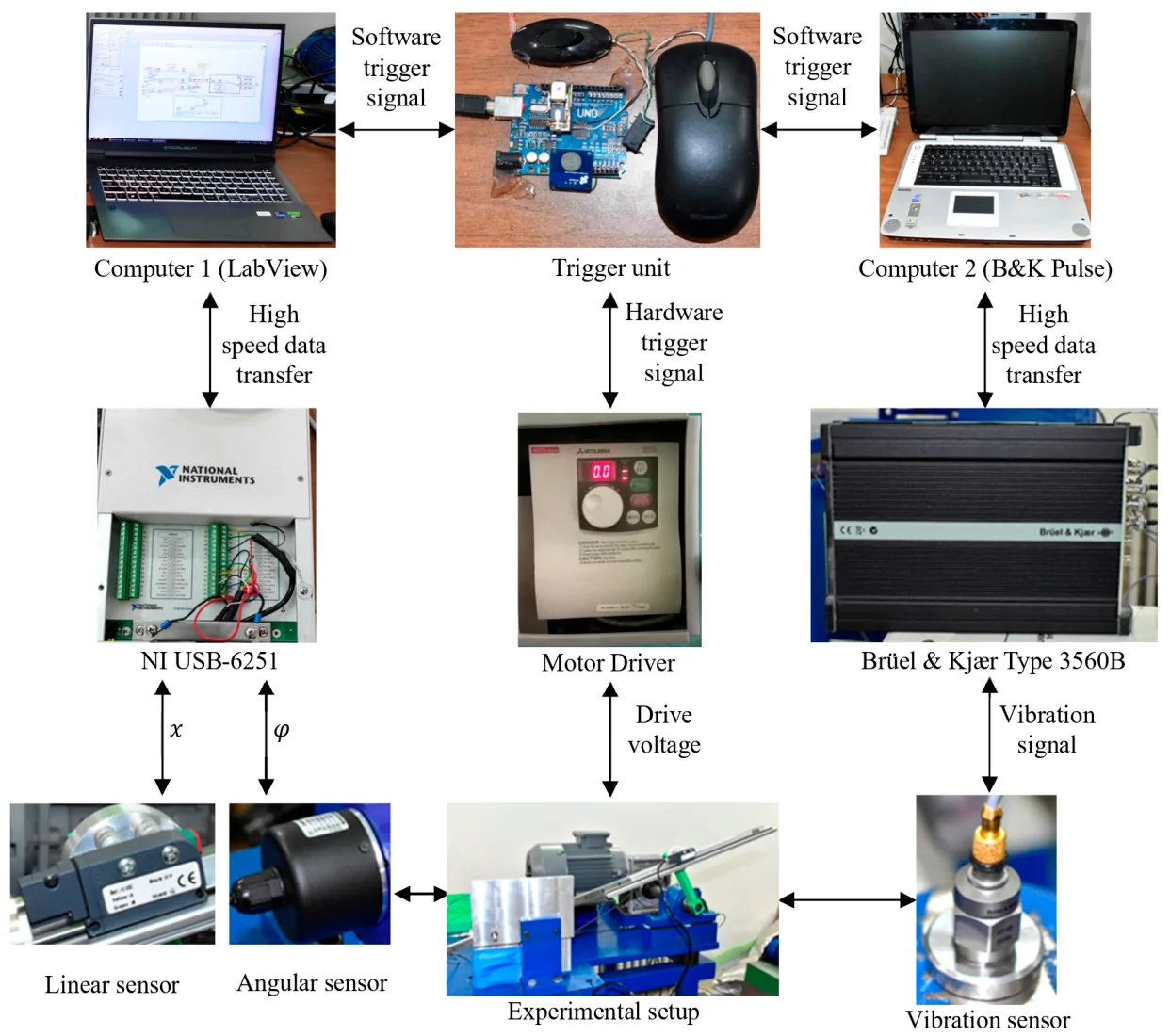
Schematic overview of the experimental system.

**Figure 5:**
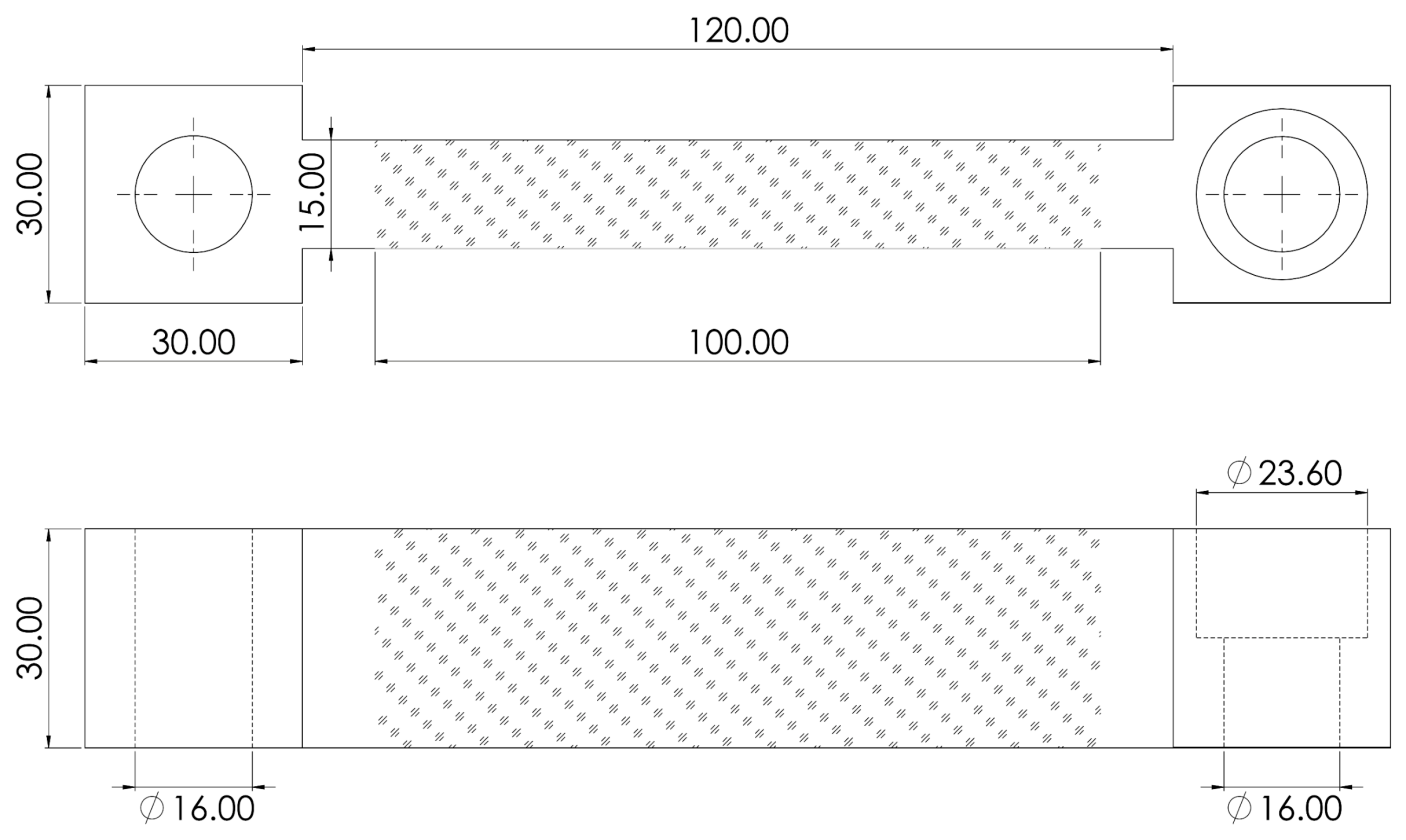
Dimensional parameters of the crank.

**Figure 6:**
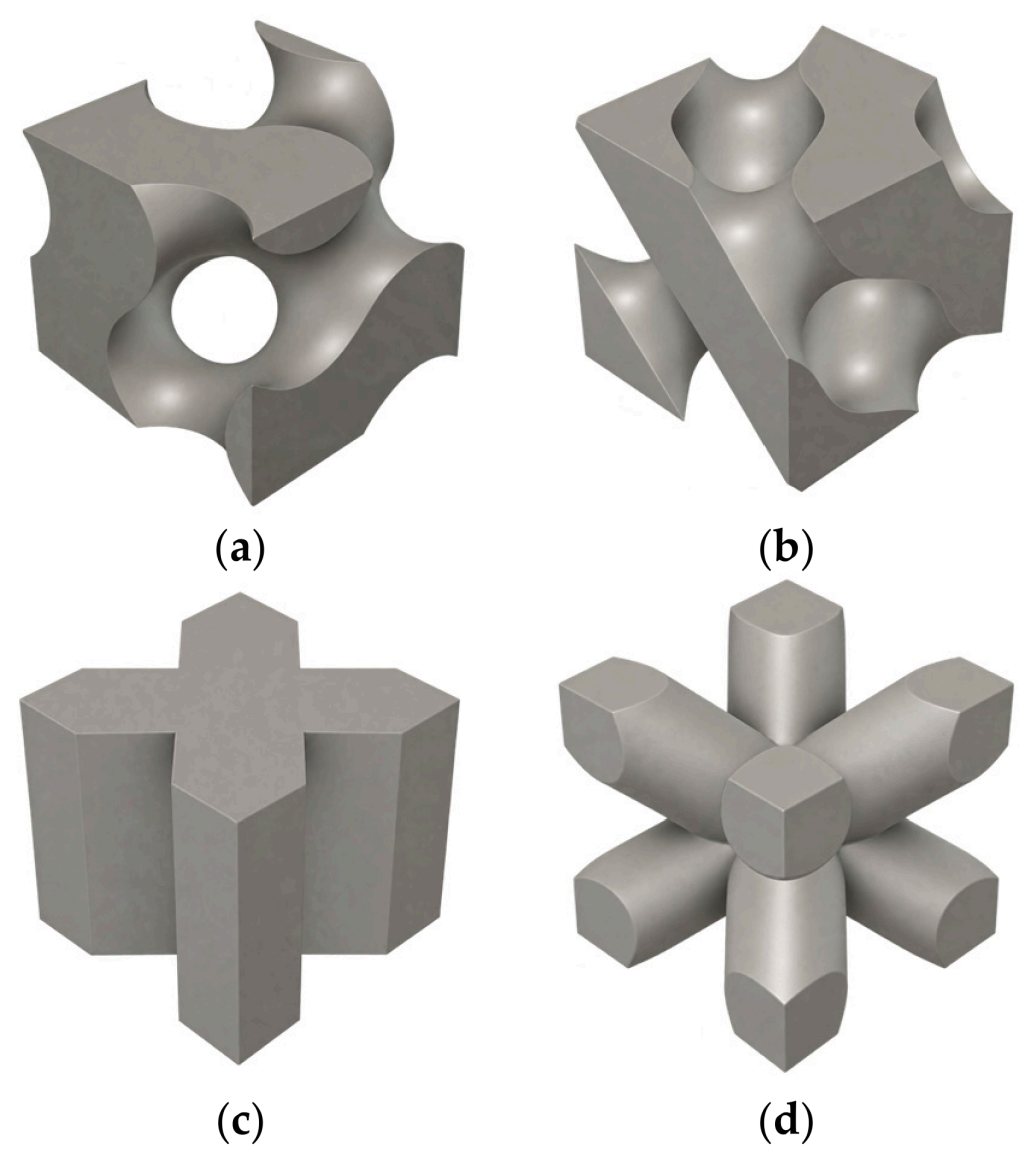
Unit-cell geometries of the investigated lattice topologies: (**a**) Gyroid, (**b**) Schwarz-D, (**c**) 2.5X-structure, and (**d**) X-structure.

**Figure 7:**
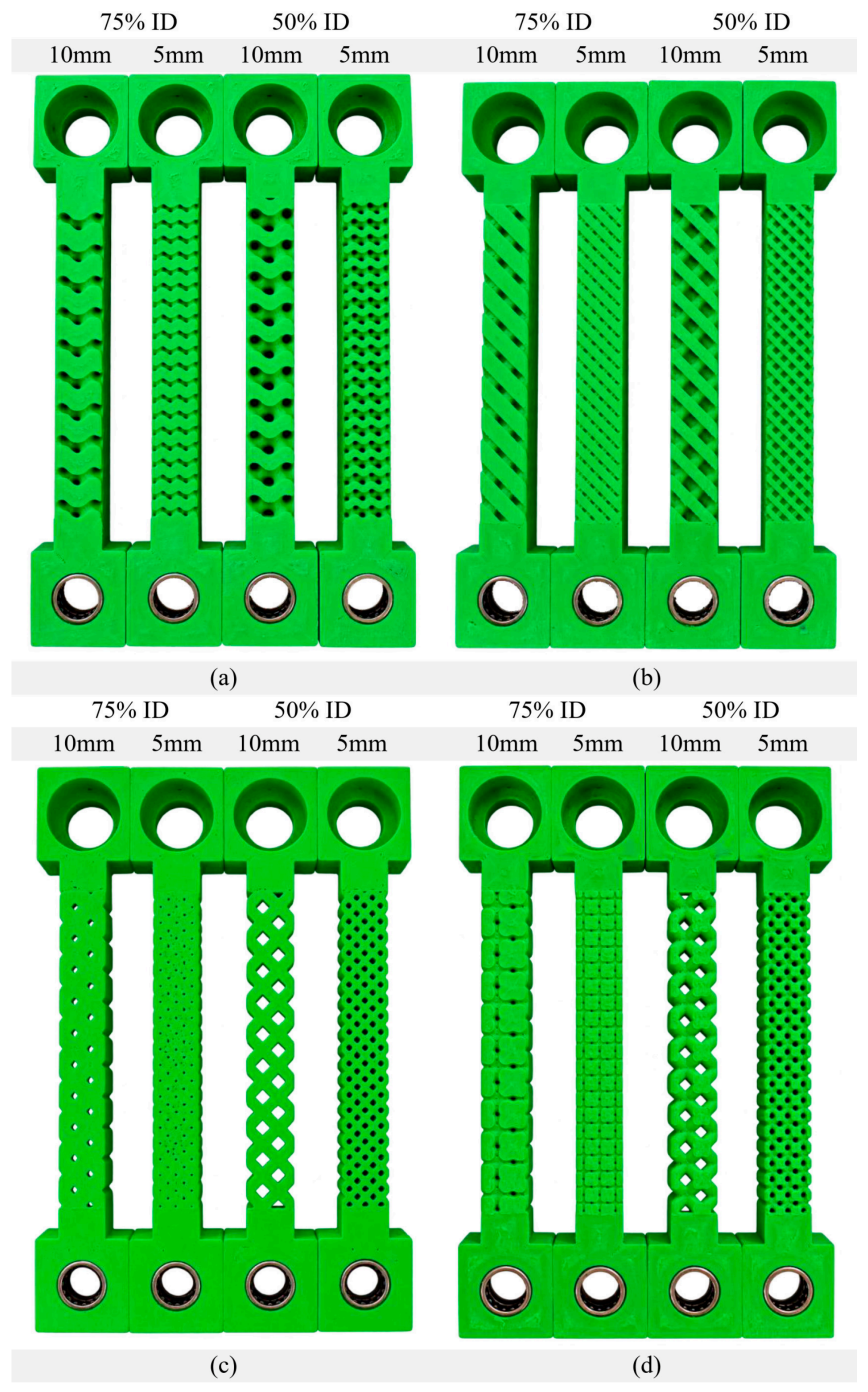
Additively manufactured lattice crank specimens (**a**) Gyroid, (**b**) Schwarz-D, (**c**) X-structure and (**d**) 2.5X-structure.

**Figure 8:**
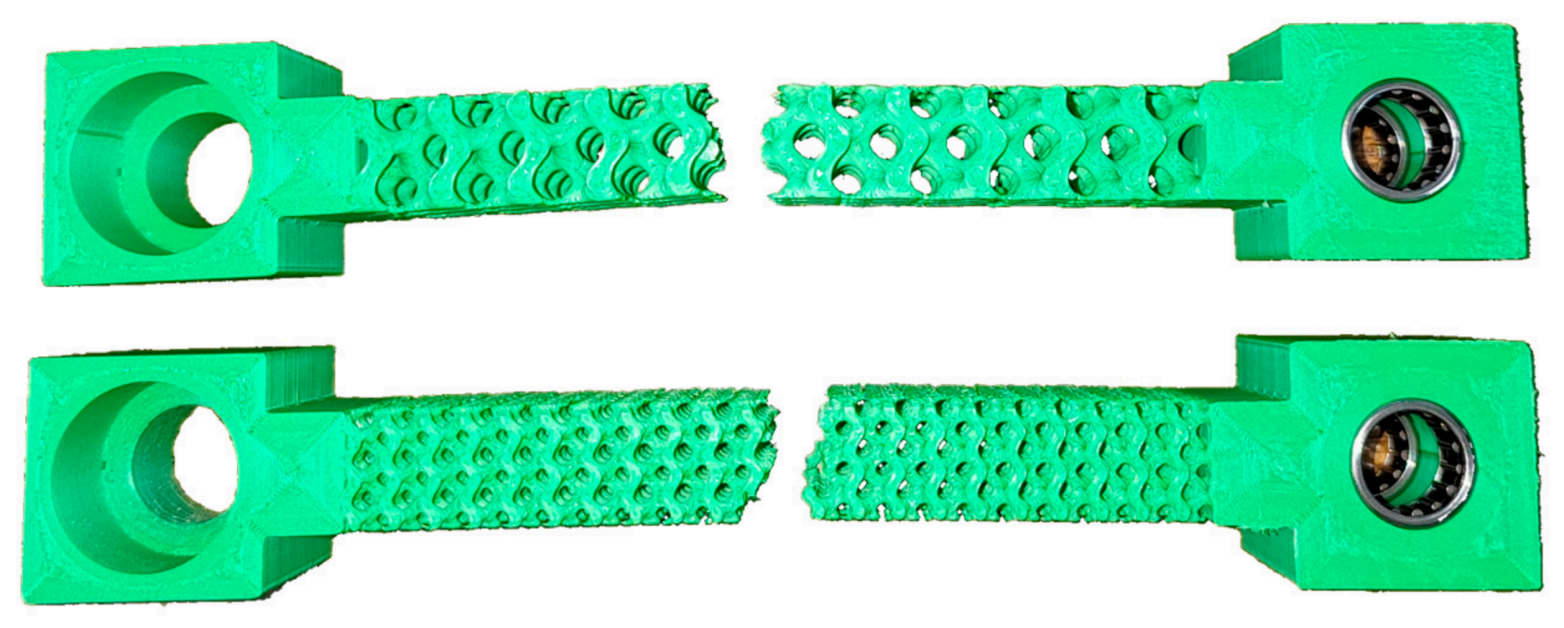
Representative fracture patterns observed in the central lattice region of the 25% ID Gyroid crank arms.

**Figure 9:**
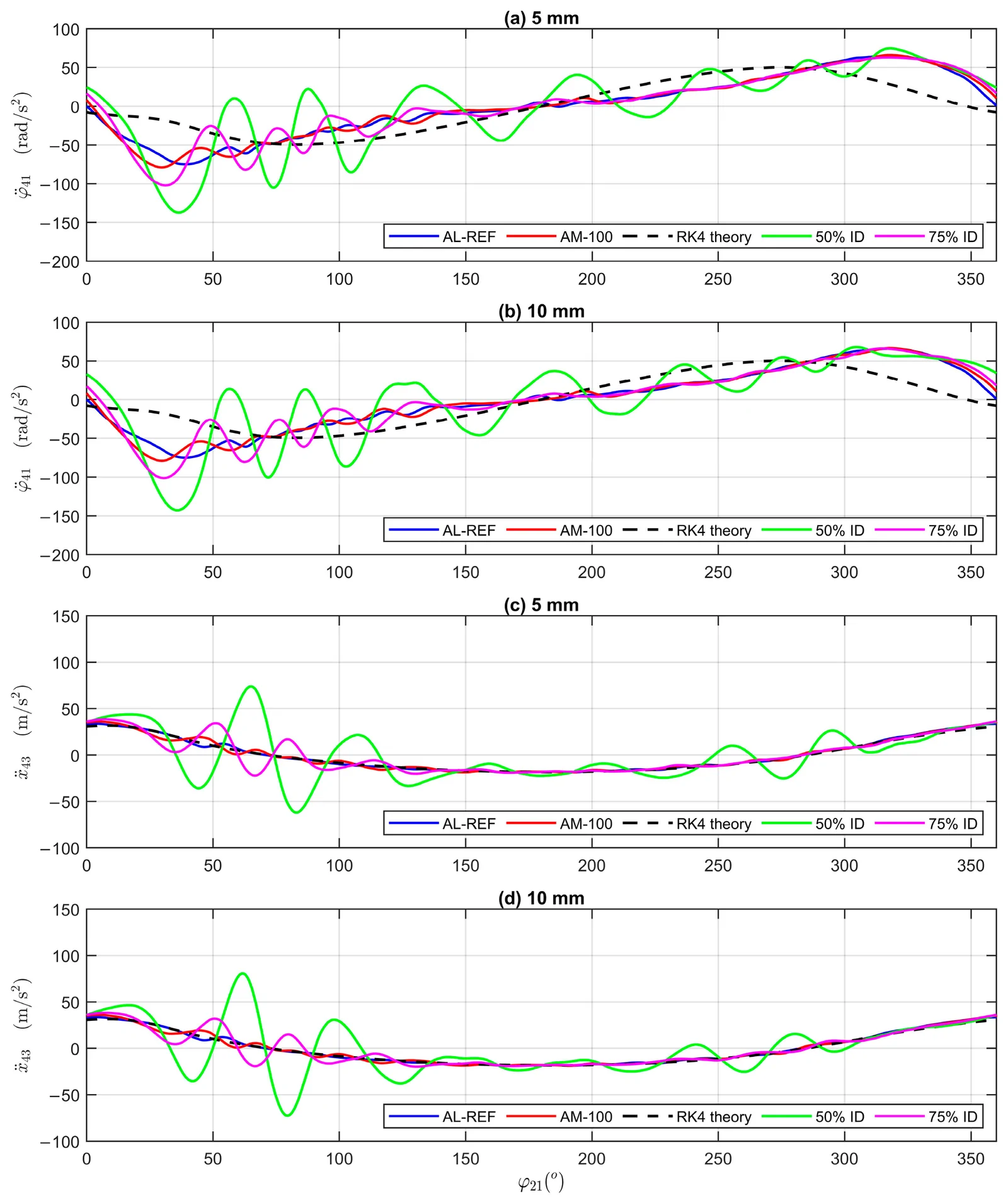
Kinematic acceleration responses of Gyroid lattice crank arms with different CSs and IDs: (**a**,**b**) output-link angular acceleration and (**c**,**d**) relative slider acceleration.

**Figure 10:**
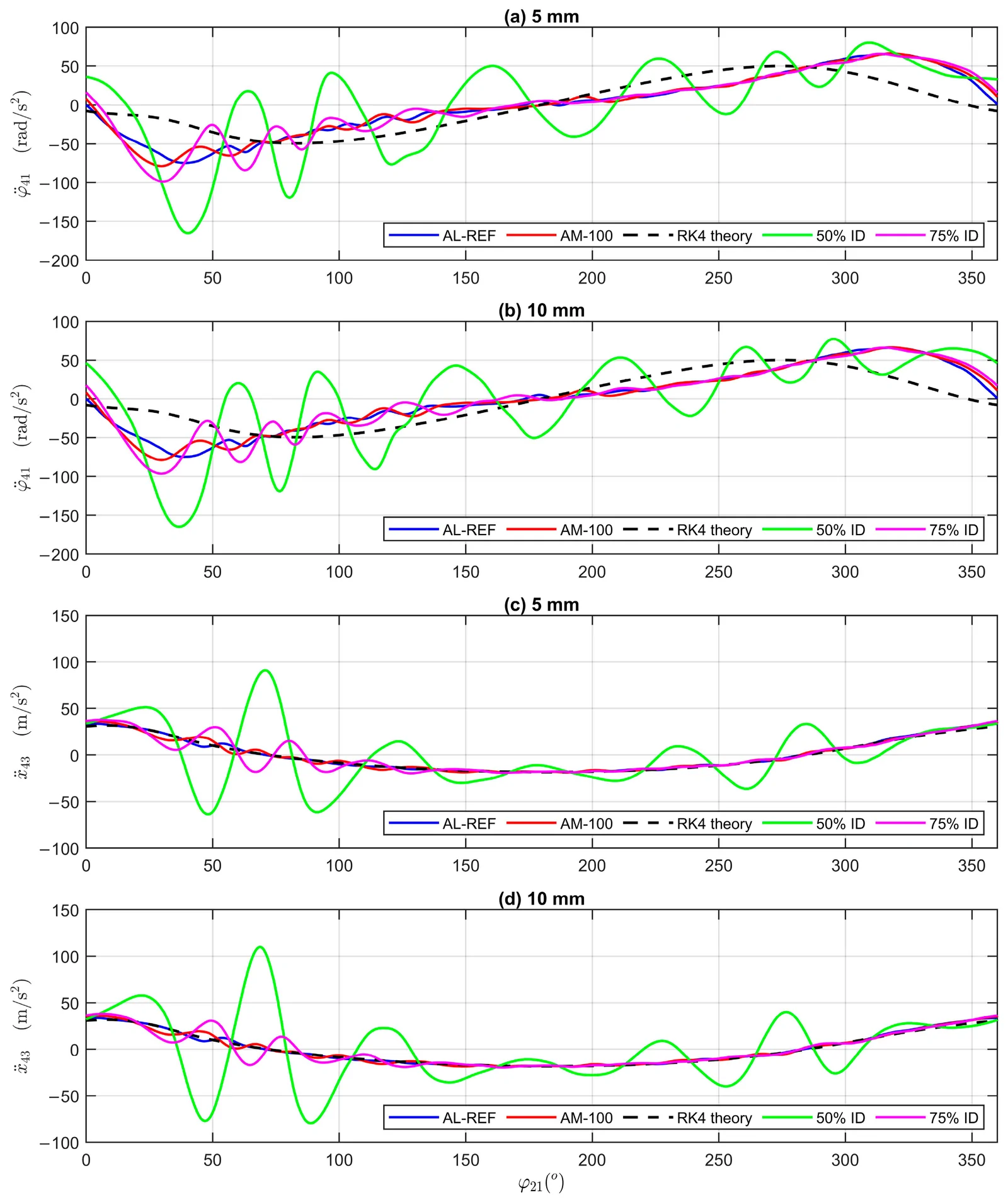
Kinematic acceleration responses of Schwarz-D lattice crank arms with different CSs and IDs: (**a**,**b**) output-link angular acceleration and (**c**,**d**) relative slider acceleration.

**Figure 11:**
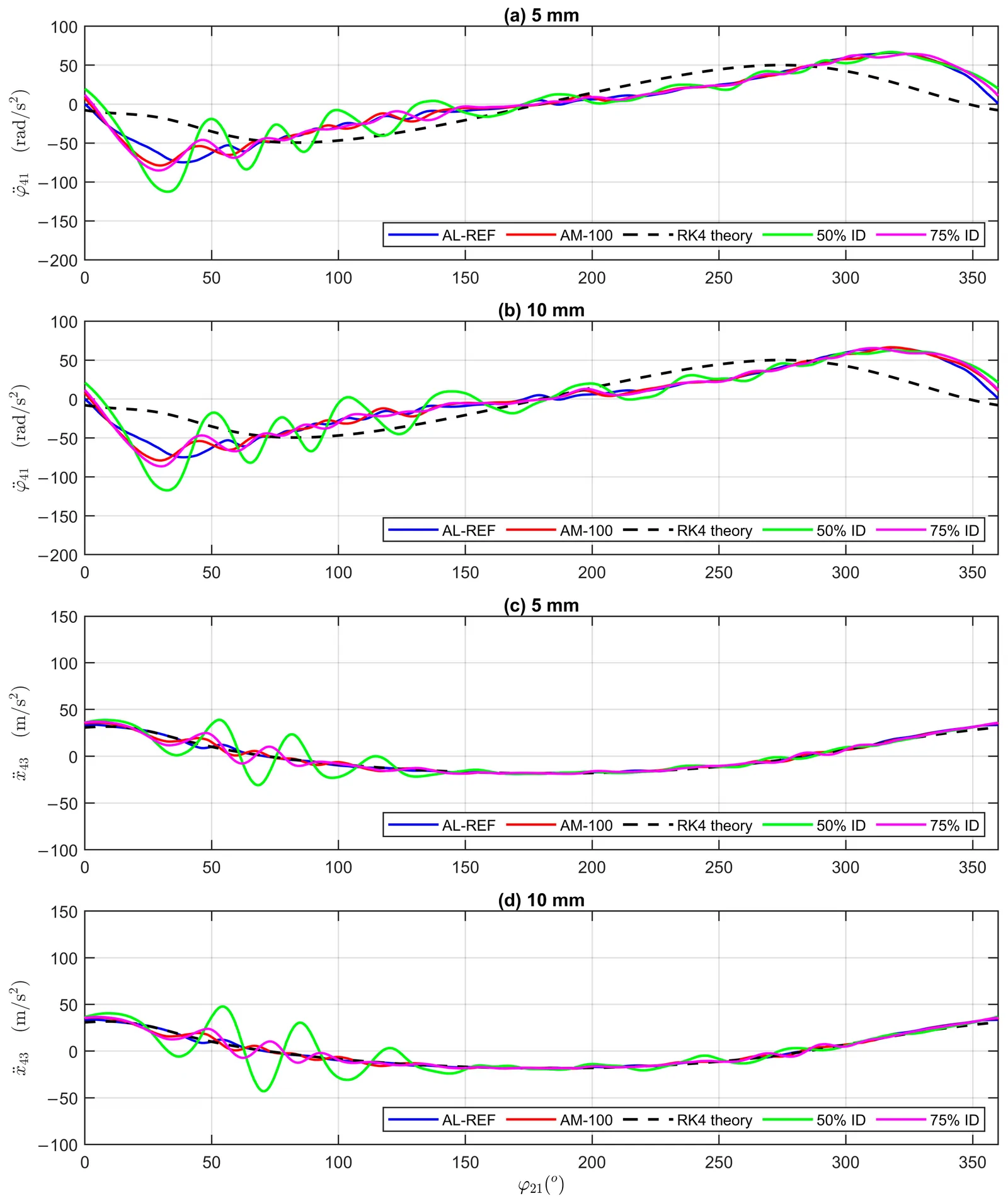
Kinematic acceleration responses of 2.5X-structure lattice crank arms with different CSs and IDs: (**a**,**b**) output-link angular acceleration and (**c**,**d**) relative slider acceleration.

**Figure 12:**
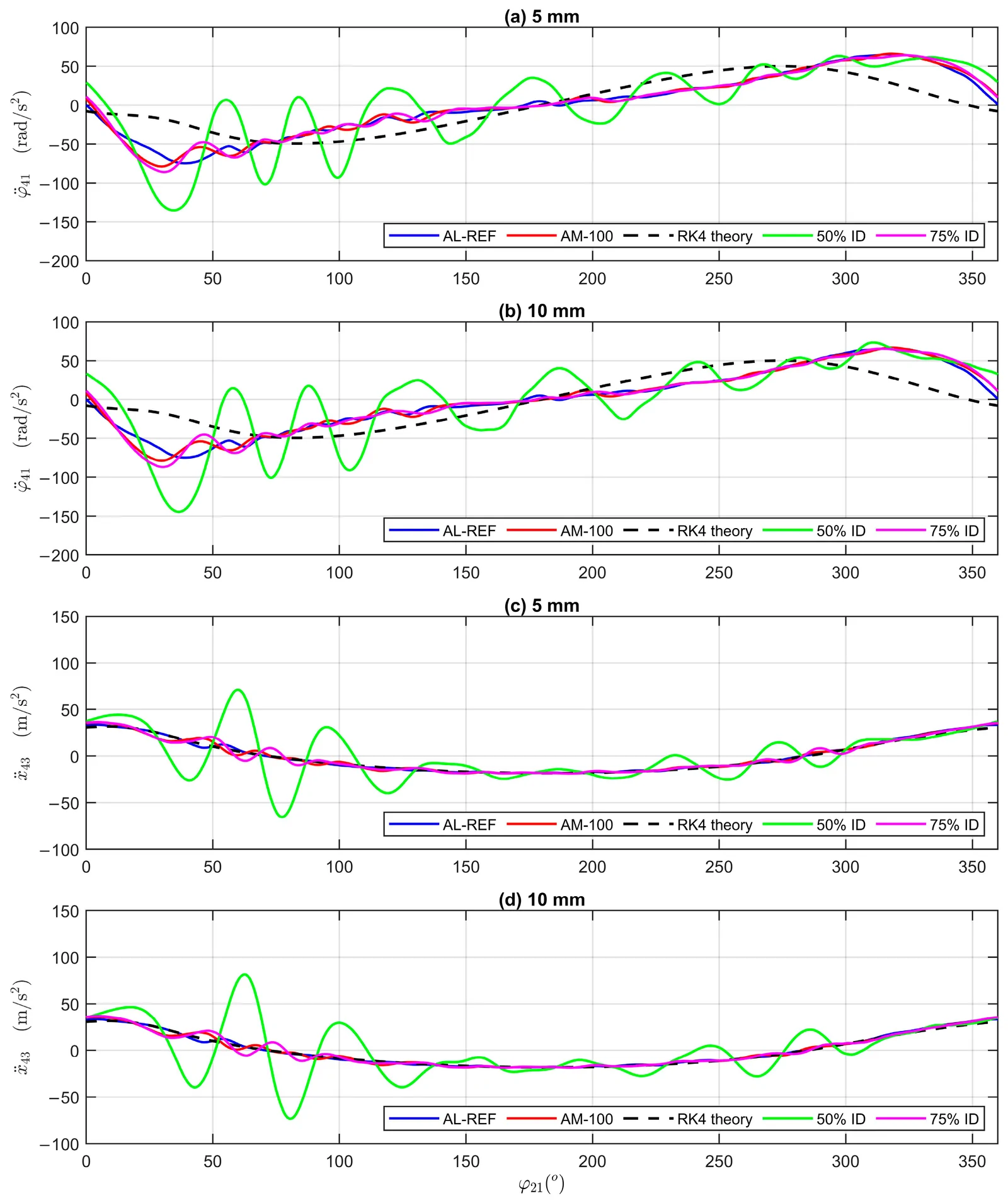
Kinematic acceleration responses of X-structure lattice crank arms with different CSs and IDs: (**a**,**b**) output-link angular acceleration and (**c**,**d**) relative slider acceleration.

**Figure 13:**
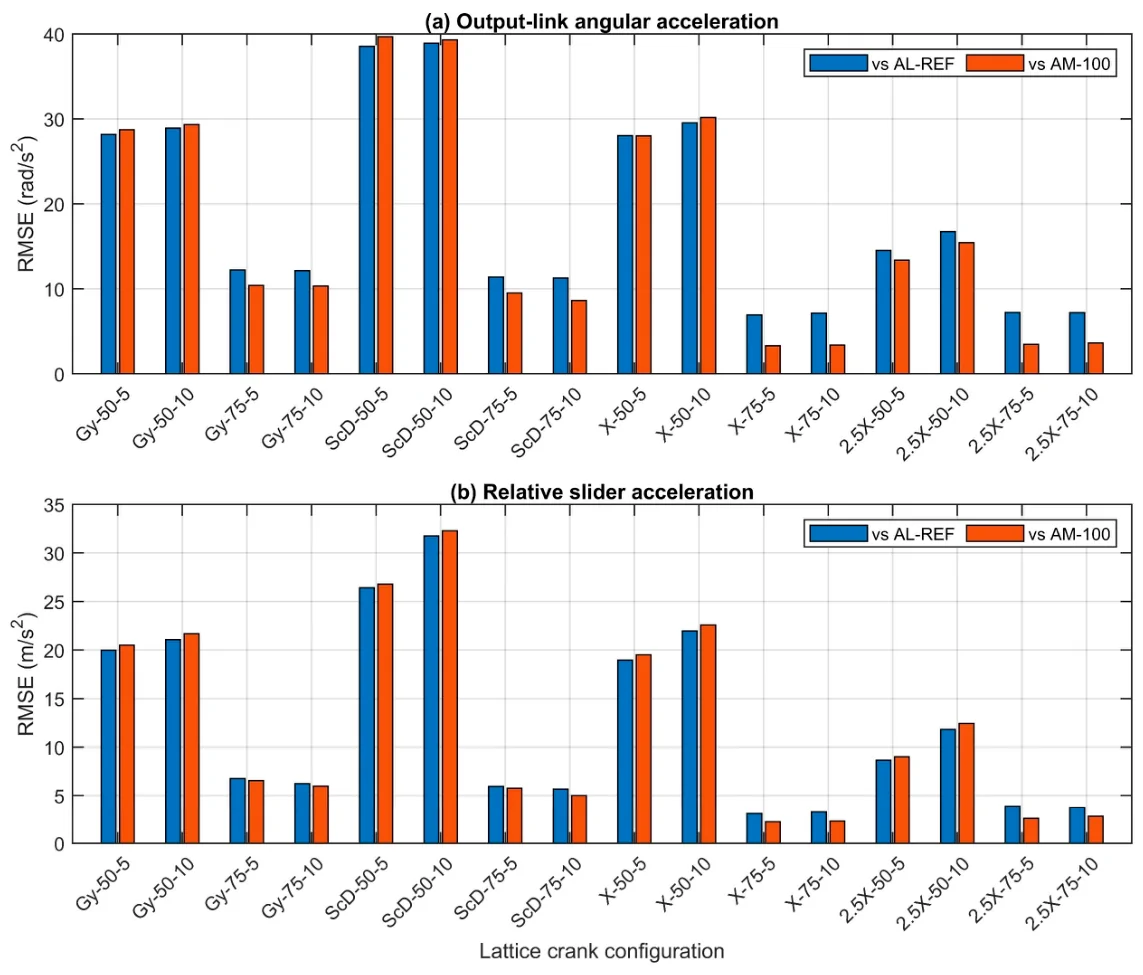
RMSE-based comparison of the kinematic acceleration responses of the lattice crank configurations with the experimental reference responses: (**a**) output-link angular acceleration and (**b**) relative slider acceleration.

**Figure 14:**
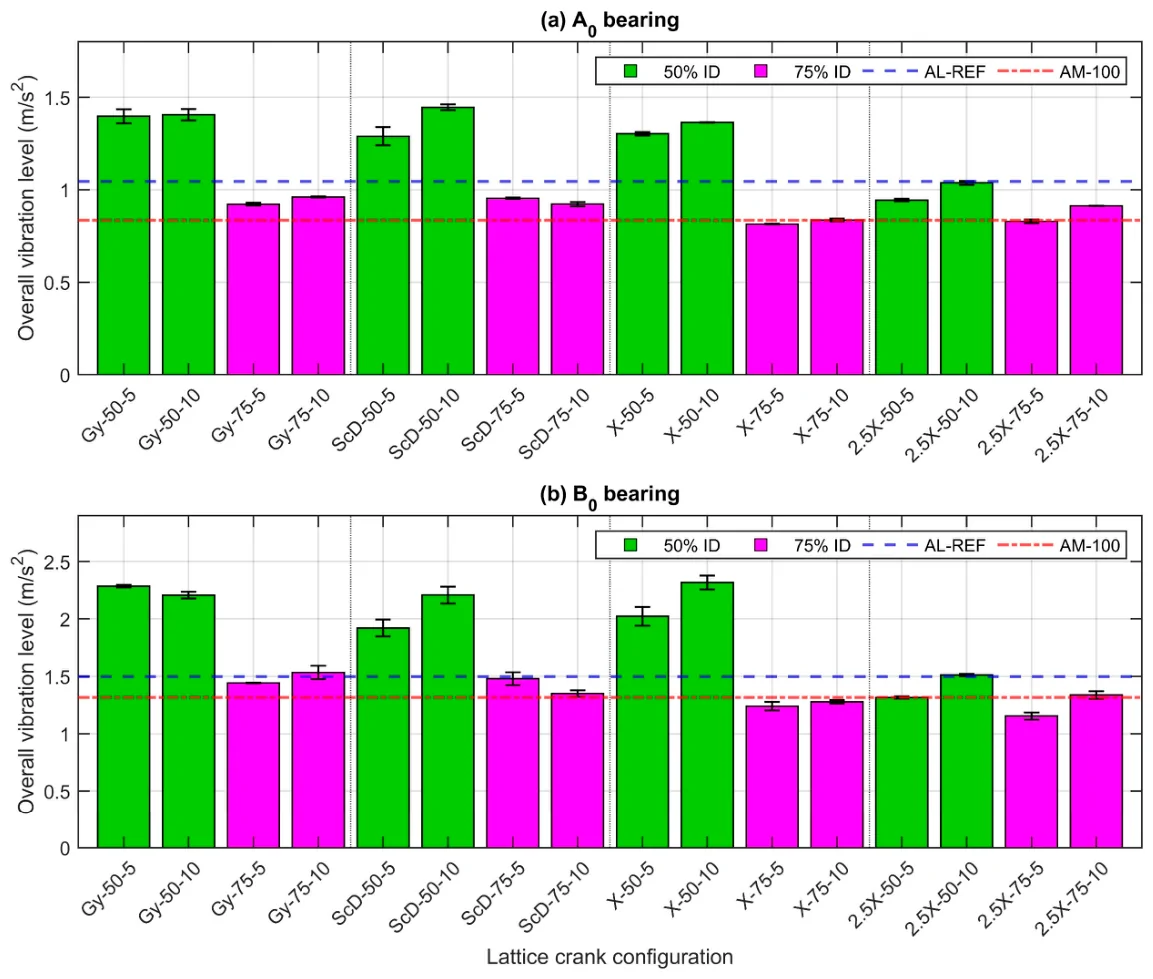
Vibration levels at the (**a**) $A_{0}$ and (**b**) $B_{0}$ bearings for different lattice crank configurations.

**Figure 15:**
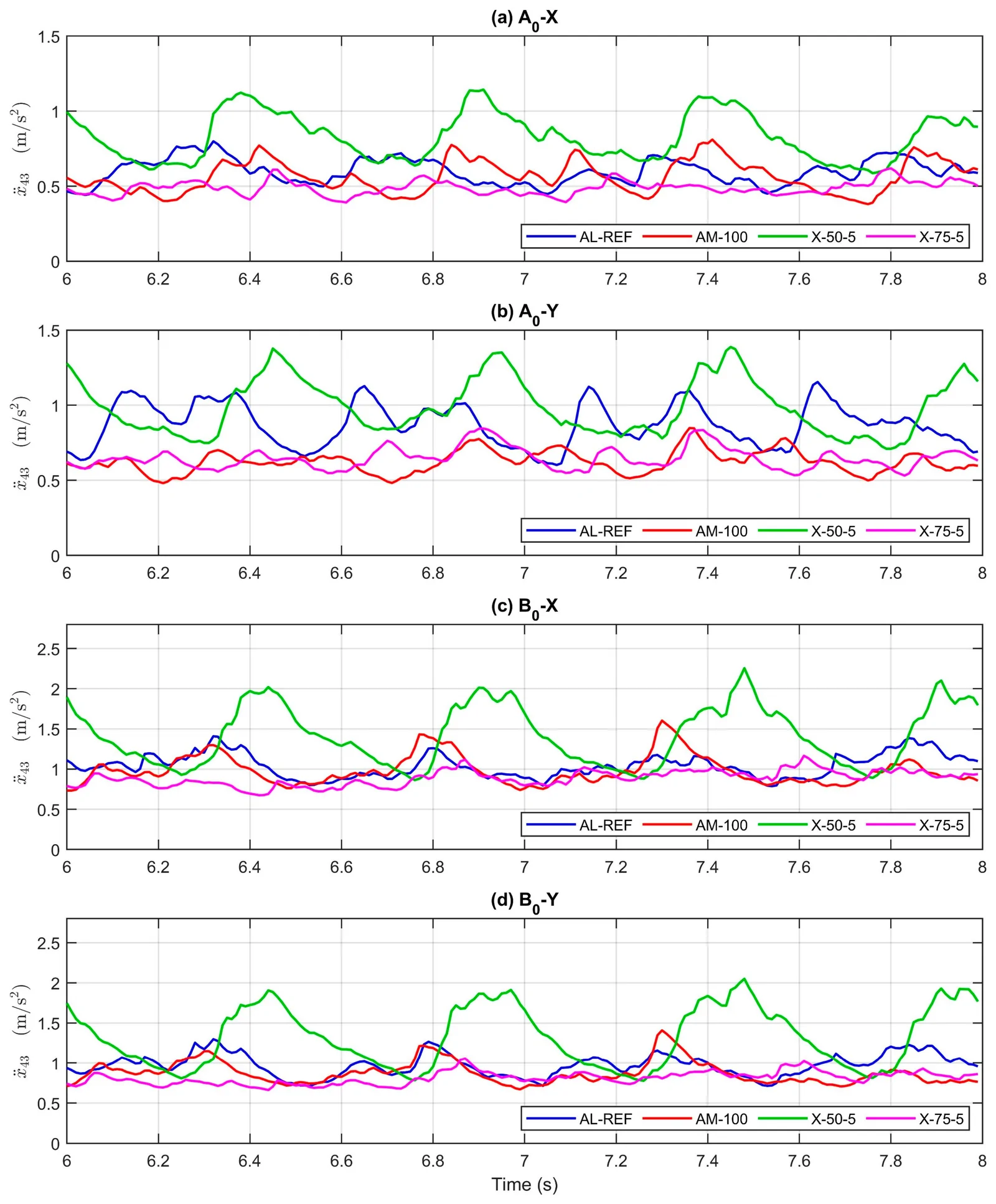
Vibration responses at the $A_{0\;}$ and $B_{0}$ bearings for the AL-REF, AM-100, X-50-5, and X-75-5 crank configurations: (**a**) $A_{0_{x}}$, (**b**) $A_{0_{y}}$, (**c**) $B_{0_{x}}$, and (**d**) $B_{0_{y}}.$

**Figure 16:**
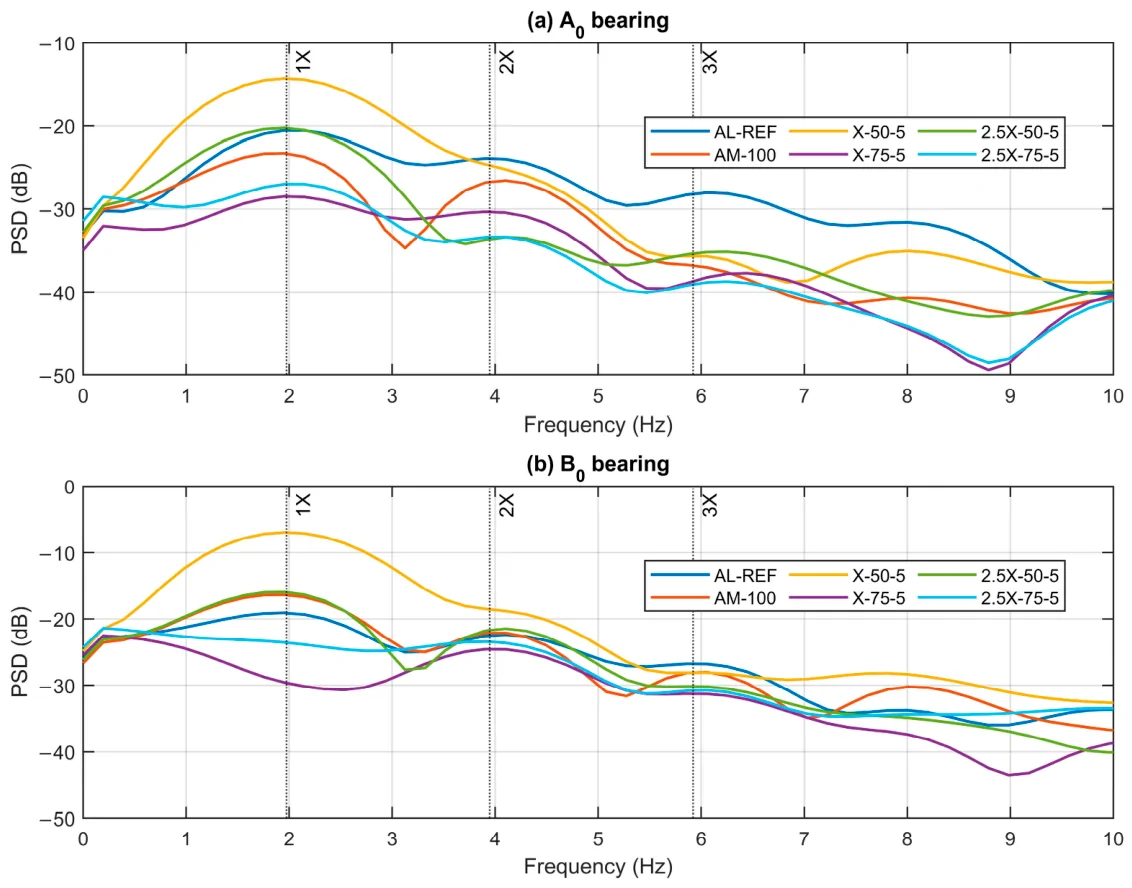
Power spectral density responses at the (**a**) $A_{0}$ and (**b**) $B_{0}$ bearings for selected crank configurations.

**Figure 17:**
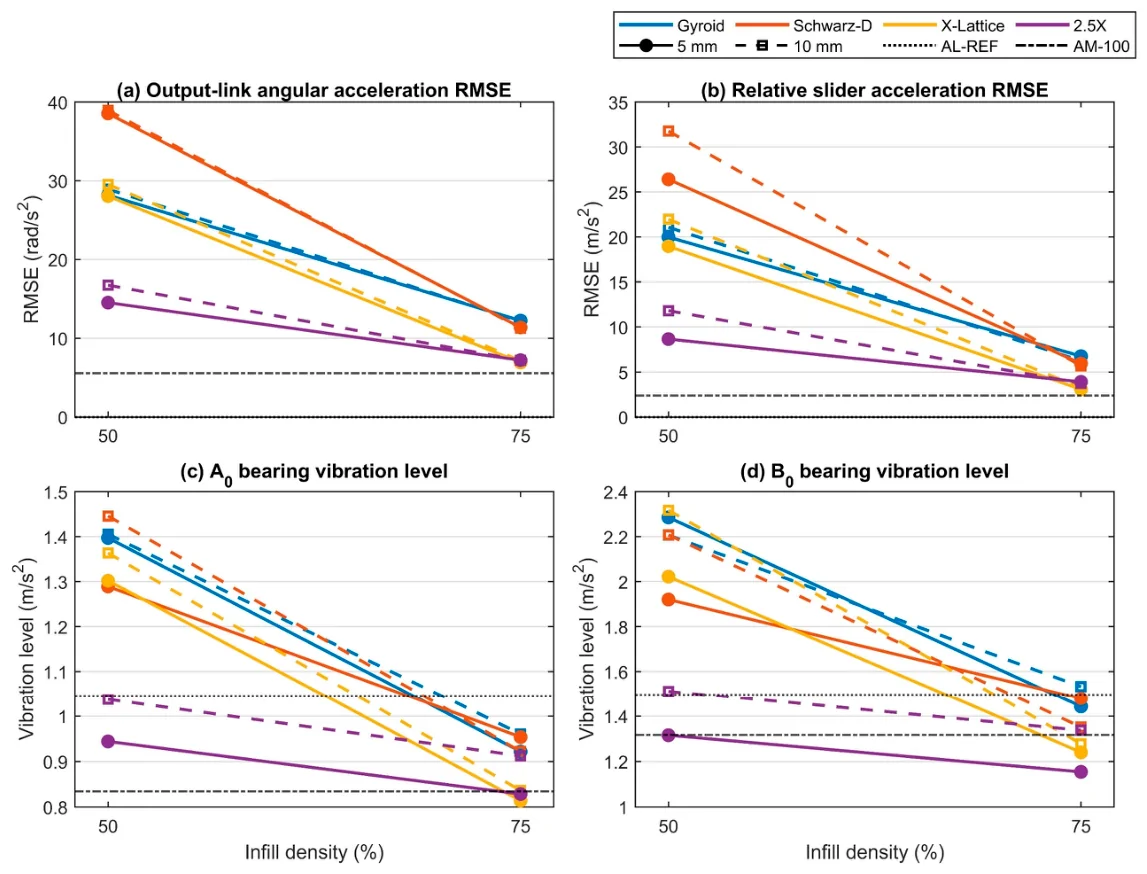
Effects of ID, lattice topology, and CS on the combined kinematic and vibration performance: (**a**) output-link angular acceleration RMSE, (**b**) relative slider acceleration RMSE, (**c**) $A_{0}$ bearing vibration level, and (**d**) $B_{0}$ bearing vibration level. Kinematic RMSE values are calculated relative to the AL-REF.

**Table 1:** Dimensional and inertial properties of the inverted slider-crank mechanism used in this study.

Length (mm)				Mass (kg)			Moment of Inertia (kg·m^2^)		
$A_{0}A$	$A_{0}G_{2}$	$A_{0}B_{0}$	$B_{0}G_{4}$	$m_{2}$	$m_{3}$	$m_{4}$	$I_{G_{2}}$	$I_{G_{3}}$	$I_{G_{4}}$
150	59.06	500	331.874	0.2537	0.4194	1.150	0.000807	0.000155	0.05965

**Table 2:** Input parameters and evaluated output quantities.

Parameter Group	Parameter	Level/Evaluation
Input parameters	Lattice topology	Gyroid, Schwarz-D, X, 2.5X
	Cell size	5 mm, 10 mm
	Infill density	25%, 50%, 75% *
Output parameters	Output link angular acceleration	${\ddot{\varphi }}_{41}$
	Slider relative linear acceleration	${\ddot{x}}_{43}$
	Kinematic deviation	RMSE relative to AL-REF and AM-100
	$A_{0}$ bearing vibration	Resultant vibration level from X–Y measurements
	$B_{0}$ bearing vibration	Resultant vibration level from X–Y measurements
	Frequency-domain vibration response	PSD; 1X, 2X, and 3X components

* Comparative kinematic and vibration analyses were conducted at the 50% and 75% ID levels, as the 25% ID specimens failed before reaching steady-state operation.

**Table 3:** Material extrusion parameters and manufacturer-reported PLA properties.

Toolpath strategy	Wall paths only *	Nozzle temperature	220 °C
Layer height	0.2 mm	Bed temperature	60 °C
Line width	0.4 mm	Printing speed	200 mm/s
Density **	1.24 g/cm^3^	Tensile strength **	51 MPa
Flexural strength **	86 MPa		

* A sufficiently high wall count was specified in the slicing software so that the material regions of the CAD models were printed entirely using wall paths, without separate infill paths. ** Density, tensile strength, and flexural strength are manufacturer-reported values obtained from the CR-PLA product documentation [52], rather than measurements performed on the printed crank arms.

**Table 4:** Nominal design parameters of the investigated crank configurations, CAD volumes of the central region, geometric volume ratios, and measured crank masses.

Configuration	$V_{L}$ (mm^3^)	Geometric Volume Ratio (%)	Crank Mass (g)
Gy-50-5	22,753.709	50.56	91.6
Gy-50-10	22,801.450	50.67	91.4
Gy-75-5	33,878.651	75.29	104.5
Gy-75-10	33,907.561	75.35	104.7
ScD-50-5	22,755.279	50.57	91.8
ScD-50-10	22,755.703	50.57	91.9
ScD-75-5	36,088.002	80.20	107.6
ScD-75-10	36,043.505	80.10	107.7
X-50-5	26,036.832	57.86	95.5
X-50-10	26,011.855	57.80	95.9
X-75-5	41,716.940	92.70	114.5
X-75-10	41,654.468	92.57	114.5
2.5X-50-5	34,136.684	75.86	105.1
2.5X-50-10	34,086.088	75.75	105.4
2.5X-75-5	42,680.585	94.85	114.6
2.5X-75-10	42,642.455	94.76	114.8

**Table 5:** Mean RMSE values of the lattice crank configurations at 50% and 75% IDs relative to the experimental reference responses.

Reference	Response	50% ID Mean RMSE	75% ID Mean RMSE	Reduction
AL-REF	${\ddot{\varphi }}_{41}$	27.93 rad/s^2^	9.44 rad/s^2^	66.2%
AM-100	${\ddot{\varphi }}_{41}$	28.01 rad/s^2^	6.59 rad/s^2^	76.5%
AL-REF	${\ddot{x}}_{43}$	20.08 m/s^2^	4.83 m/s^2^	76.0%
AM-100	${\ddot{x}}_{43}$	20.60 m/s^2^	4.15 m/s^2^	79.9%

**Table 6:** Combined mass, kinematic, and vibration performance of the 75% ID lattice crank configurations.

Configuration	Mass (g)	Mass Reduction vs. AL-REF (%)	Output-Link RMSE (rad/s^2^)	Slider RMSE (m/s^2^)	($A_{0}$) Vibration (m/s^2^)	($B_{0}$) Vibration (m/s^2^)
**Gy-75-5**	104.5	58.8	12.24	6.76	0.922	1.442
**Gy-75-10**	104.7	58.7	12.15	6.25	0.960	1.532
**ScD-75-5**	107.6	57.6	11.38	5.95	0.954	1.479
**ScD-75-10**	107.7	57.5	11.29	5.68	0.922	1.351
**X-75-5**	114.5	54.9	**6.92**	**3.08**	**0.814**	**1.240**
**X-75-10**	114.5	54.9	7.14	3.25	0.836	1.278
**2.5X-75-5**	114.6	54.8	7.22	3.91	0.828	**1.154**
**2.5X-75-10**	114.8	54.7	7.20	3.75	0.912	1.337

Note: The AL-REF and AM-100 masses were 253.7 g and 118.3 g, respectively. The corresponding A_0_/B_0_ vibration levels were 1.045/1.497 m/s^2^ for AL-REF and 0.834/1.316 m/s^2^ for AM-100, respectively. The bold values indicate the best (lowest) values for the corresponding performance metrics among the 75% ID configurations.

## Data Availability

Data are available from the corresponding author upon reasonable request.

## Abbreviations

The following abbreviations are used in this manuscript:

AL-REF	Aluminum reference crank
AM	Additive manufacturing
AM-100	100% solid additively manufactured reference crank
MEX	Material extrusion
CAD	Computer-aided design
FFT	Fast Fourier transform
Gy	Gyroid
ID	Infill density
CS	Cell size
PLA	Poly(lactic acid)
PSD	Power spectral density
RK4	Fourth-order Runge–Kutta method
RMSE	Root mean square error
ScD	Schwarz-D
TPMS	Triply periodic minimal surface
NI	National Instruments
STL	Stereolithography
